# The protease‐inhibitor SerpinB3 as a critical modulator of the stem‐like subset in human cholangiocarcinoma

**DOI:** 10.1111/liv.15049

**Published:** 2021-09-16

**Authors:** Margherita Correnti, Andrea Cappon, Mirella Pastore, Benedetta Piombanti, Giulia Lori, Douglas V. P. N. Oliveira, Patricia Munoz‐Garrido, Monika Lewinska, Jesper B. Andersen, Cédric Coulouarn, Laurent Sulpice, Caterina Peraldo Neia, Giuliana Cavalloni, Santina Quarta, Alessandra Biasiolo, Matteo Fassan, Matteo Ramazzotti, Matteo Parri, Stefania Recalcati, Luca di Tommaso, Claudia Campani, Pietro Invernizzi, Guido Torzilli, Fabio Marra, Patrizia Pontisso, Chiara Raggi

**Affiliations:** ^1^ Center for Autoimmune Liver Diseases Humanitas Clinical and Research Center Rozzano Italy; ^2^ Department of Biomedical Sciences for Health University of Milan Milan Italy; ^3^ Animal Care‐Polo Vallisneri University of Padua Padua Italy; ^4^ Department of Experimental and Clinical Medicine University of Florence Florence Italy; ^5^ Biotech Research and Innovation Centre University of Copenhagen Copenhagen Denmark; ^6^ CHU Rennes Service de Chirurgie Hépatobiliaire et Digestive Inserm Univ Rennes COSS (Chemistry, Oncogenesis Stress Signaling) UMR_S 1242 Centre de Lutte contre le Cancer Eugène Marquis Rennes France; ^7^ CHU Rennes Service de Chirurgie Hépatobiliaire et Digestive INSERM 1241 Université de Rennes Rennes France; ^8^ Cancer Genomics Lab Fondazione Edo ed Elvo Tempia Valenta Biella Italy; ^9^ Division of Medical Oncology Candiolo Cancer Institute FPO‐IRCCS Candiolo, Torino Italy; ^10^ Department of Medicine‐DIMED University of Padua Padua Italy; ^11^ Department of Experimental and Clinical Biomedical Sciences University of Florence Florence Italy; ^12^ Department of Pathology Humanitas Clinical and Research Center Rozzano Italy; ^13^ Department of Biomedical Sciences Humanitas University Rozzano Italy; ^14^ Division of Gastroenterology and Center for Autoimmune Liver Diseases Department of Medicine and Surgery University of Milano‐Bicocca Monza Italy; ^15^ European Reference Network on Hepatological Diseases (ERN RARE‐LIVER) San Gerardo Hospital Monza Italy; ^16^ Department of Hepatobiliary and General Surgery Humanitas University Humanitas Clinical and Research Center IRCCS, Rozzano Milan Italy

**Keywords:** cancer stem cells, cholangiocarcinoma, invasion, SerpinB3

## Abstract

**Background and aims:**

Cholangiocarcinoma (CCA) is a form of primary liver cancer with limited therapeutic options. Recently, cancer stem cells (CSCs) have been proposed as a driving force of tumour initiation and dissemination, thus representing a crucial therapeutic target. The protease inhibitor SerpinB3 (SB3) has been identified in several malignancies including hepatocellular carcinoma. SB3 has been involved in the early events of hepatocarcinogenesis and is highly expressed in hepatic progenitor cells and in a mouse model of liver progenitor cell activation. However, only limited information on the possible role of SB3 in CCA stem‐like compartment is available.

**Methods:**

Enrichment of CCA stem‐like subset was performed by sphere culture (SPH) in CCA cell lines (CCLP1, HUCCT1, MTCHC01 and SG231). Quantitative RT‐PCR and Western blotting were used to detect SB3 in both SPH and parental monolayer (MON) cells. Acquired CSC‐like features were analysed using an endogenous and a paracrine in vitro model, with transfection of SB3 gene or addition of recombinant SB3 to cell medium respectively. SB3 tumorigenic role was explored in an in vivo mouse model of CCA by subcutaneous injection of SB3‐transfected MON (MON^SB3+^) cells in immune‐deficient NOD‐SCID/*IL2Rg* ^null^ (NSG) mice. SB3 expression in human CCA sections was investigated by immunohistochemistry. Overall survival (OS) and time to recurrence (TTR) analyses were carried out from a transcriptome database of 104 CCA patients.

**Results:**

SB3, barely detected in parental MON cells, was overexpressed in the same CCA cells grown as 3D SPH. Notably, MON^SB3+^ showed significant overexpression of genes associated with stemness (CD24, CD44, CD133), pluripotency (c‐MYC, NOTCH1, STAT3, YAP, NANOG, BMI1, KLF4, OCT4, SOX2), epithelial mesenchymal transition (β‐catenin, SLUG) and extracellular matrix remodelling (MMP1, MMP7, MMP9, ADAM9, ADAM10, ADAM17, ITGB3). SB3‐overexpressing cells showed superior spherogenic capacity and invasion ability compared to control. Importantly, MON^SB3+^ exhibited activation of MAP kinases (ERK1/2, p38, JNK) as well as phosphorylation of NFκB (p65) in addition to up‐regulation of the proto‐oncogene β‐catenin. All these effects were reversed after transient silencing of SB3. According to the in vitro finding, MON^SB3+^ cells retained high tumorigenic potential in NSG mice. SB3 overexpression was observed in human CCA tissues and analysis of OS as well as TTR indicated a worse prognosis in SB3^+^ CCA patients.

**Conclusion:**

These findings indicate a SB3 role in mediating malignant phenotype of CCA and identify a new therapeutic target.

**Abbreviations**
CCA: Cholangiocarcinoma
CSC: Cancer Stem Cells
SB3: SerpinB3

**Lay Summary**

Biomarker approach to screen cholangiocarcinoma cancer stem cells has gained impetus since the past decade. Progress in the identification and characterization of the stem cell biomarkers has led to many insights. Our study provides evidence for serine‐protease SerpinB3 as a new molecule involved in the biology of the cholagiocarcinoma stem‐like compartment thus mediating the malignant phenotype of this malignancy. Such a study holds the potential to predict therapeutic response and clinical outcome in patients.

## 1 INTRODUCTION

Cholangiocarcinoma (CCA) is a primary liver cancer of biliary epithelial cells, and together with hepatocellular carcinoma (HCC) represents a major form of hepatic malignancy. CCA is considered one of the deadliest types of cancer, and its aetiopathogenesis remains largely unknown.^1^, ^2^, ^3^ CCA incidence is constantly increasing in Europe and its mortality has been rising worldwide in the last decades.^1^, ^2^, ^3^ Identification of cancer stem cells (CSCs) has opened the way to design innovative diagnostic and therapeutic strategies in neoplastic diseases. CSCs, also referred to as tumour‐initiating cells or tumour‐propagating cells, are tumorigenic, metastatic, resistant to chemo‐ and radiation therapy and are responsible for tumour recurrence. Recently, we and others have provided data indicating CSCs as a driving force of CCA initiation, dissemination and drug‐resistance.^4^, ^5^, ^6^, ^7^, ^8^, ^9^, ^10^, ^11^, ^12^, ^13^, ^14^, ^15^, ^16^

SerpinB3 (formerly known as squamous cell carcinoma antigen‐1 or SCCA1) is a member of the serine‐protease inhibitors family.^17^ It is expressed in normal epithelial cells and overexpressed in cancer cells and damaged hepatocytes.^18^, ^19^, ^20^, ^21^ High levels of SerpinB3 (SB3) are correlated with molecular markers of poor prognosis, including TGF‐β signalling and Wnt target genes expression (ie β‐catenin activation), and induce epithelial mesenchymal transition (EMT), increased invasiveness and cell proliferation in liver cancer cells.^17^ Moreover, c‐Myc expression has been shown to be up‐regulated by SB3 through calpain and Hippo‐dependent molecular mechanisms.^22^ In the liver, SB3 and its related isoforms are undetectable in normal hepatocytes, but their expression progressively increases in chronic liver diseases, dysplastic nodules and HCC.^23^, ^24^, ^25^, ^26^ High SB3 levels have been recently detected in HCC tissue of patients with early tumour recurrence after surgical resection, suggesting their involvement in liver malignancy.^23^, ^24^, ^25^, ^26^

SB3 is expressed in human liver progenitor cells,^27^ as indicated by its detection in EpCAM^+^ cell fractions sorted from human foetal and adult livers and in ductular liver structures expressing stem/progenitor cell markers. Furthermore, the mouse homologous, *SerpinB3b*, was induced by the administration of lipopolysaccharide and D‐galactosamine, a model of liver stem/progenitor cell activation. SB3 overexpression was also observed in hepatoblastoma, especially in its embryonic, blastemal and small cell undifferentiated components.^28^ Based on the highly pro‐oncogenic potential of SB3 together with its significant expression in human liver progenitor cells, this study aimed to explore the role of this serine‐protease in the biology of the CCA stem‐like compartment.

## 2 RESULTS

### 2.1 Expression levels and biological effects of SB3 in the CCA stem‐like subset

**FIGURE 1:**
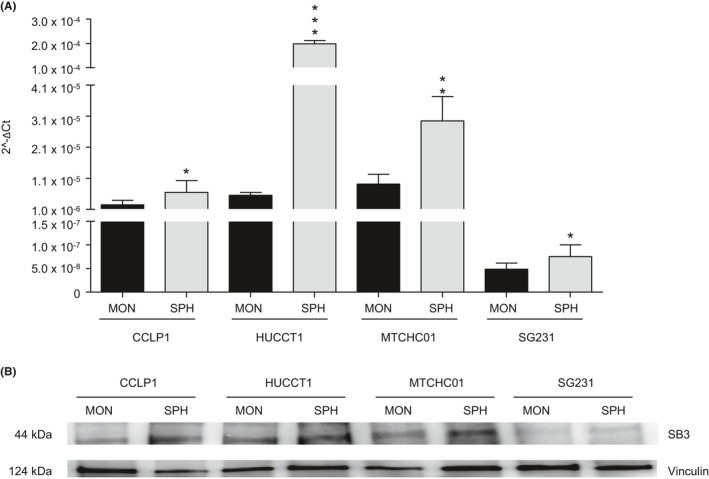
(A) Relative expression of transcript‐encoding SerpinB3 (SB3) in sphere (SPH) and monolayer (MON) of CCLP1, HUCCT1, MTCHC01 and SG231 cells. GAPDH was used as the internal control. All mRNA levels are presented as 2^−ΔCt. Data are expressed as mean ±SEM (*P* value vs MON by Student t test, *P ≤ .05, **P ≤ .01, ***P ≤ .001). (B) Western blot analysis was used to determine SB3 in CCLP1, HUCCT1, MTCHC01 and SG231 cells. Equal loading was evaluated using anti‐Vinculin antibody

We recently identified a functional CSC‐subset in human CCA by using a 3D sphere (SPH) culture model.^10^ To understand whether SB3 may have a role in the biology of CCA stemness compartment, the presence of this serine‐protease inhibitor was first investigated in established human intrahepatic CCA (iCCA) cell lines (CCLP1, HUCCT1 MTCHC01, SG231) cultured as an adherent monolayer (MON) or 3D SPH. Consistent with their superior tumour stem‐like properties,^10^ SB3 expression was markedly increased at the protein and mRNA levels in CCA‐SPH compared to MON in both cell lines (Figure 1A,B). It is important to note that although HUCCT1 and MTCHC01 SPH showed very similar SB3 protein content, differences at the mRNA level were enormous (Figure 1A,B) thus suggesting a possible post‐transcriptional regulation.

Based on these initial results, we next evaluated whether SB3 could have a role in the modulation of the CCA stem‐like features. To address this issue, we decided to focus on HUCCT1 and SG231 cells which were well‐characterized in terms of stem‐like features by our group.^10^ Together with these, we decided to use another CCA cell line available in our laboratory, the MTCHC01, which has not yet been extensively characterized in terms of stemness features as for the others (HUCCT1, SG231).^10^ Nevertheless, a marked expression for several stem‐related genes of MTCHC01 SPH compared to MONs was revealed by qRT‐PCR Array (Figure S1). This result further confirmed that, as for the other previously studied CCA cells, the 3D SPH culture system does represent a good tool to select and enrich for CSCs.

Importantly two different in vitro approaches were used (Figure S2): (a) CCA MON cells stably transfected with SB3 expression vectors (MON^SB3+^) were used to evaluate the role of endogenous over‐production of this factor; (b) 6 days cultures of MON in the presence of 150 ng/mL of human recombinant SB3 (+rhSB3) were used to investigate the paracrine effect of the exogenously added molecule. Based on preliminary data (data not shown), in our experimental setting the amount of SB3 used for the exogenous treatment (150 ng/mL) was about 50‐ to 30‐folds higher than that excreted by SPH cells.

**FIGURE 2:**
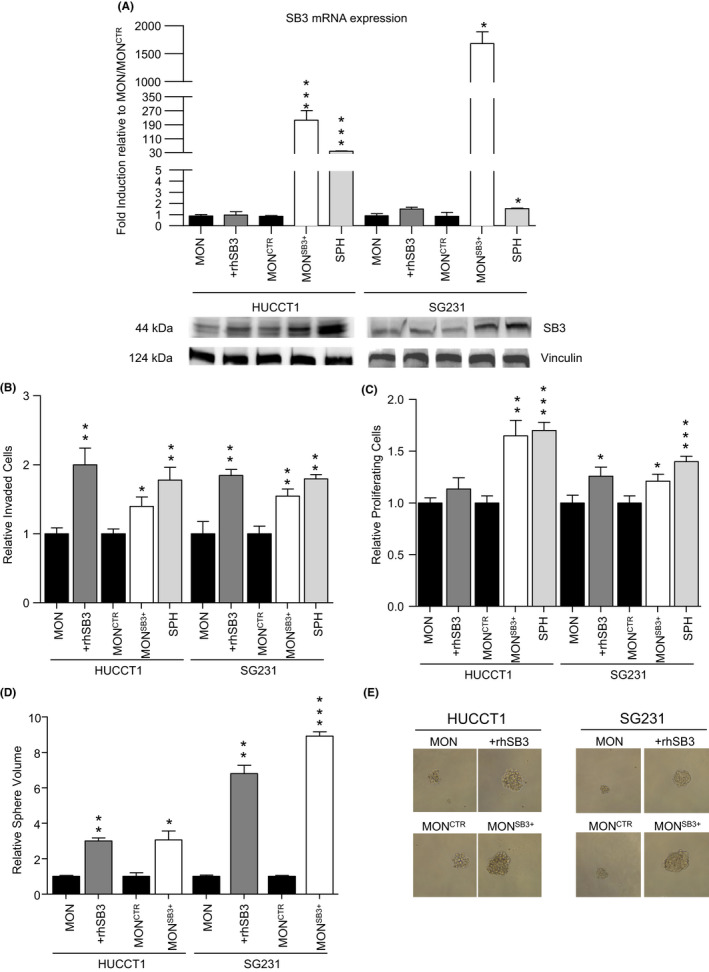
(A) Comparison of paracrine (+rhSB3) and endogenous (MON^SB3+^) effect on the expression of SB3 (SB3) mRNA in HUCCT1 and SG231 cells. GAPDH was used as the internal control. Not treated monolayer (MON), monolayer transfected with control vector (MON^CTR^) as controls. Sphere (SPH) condition was also evaluated. All mRNA levels are presented as fold changes normalized to 1 (mean expression of MON or MON^CTR^ respectively). Data are expressed as mean ± SEM (*P* value vs MON or MON^CTR^, respectively, by Student *t* test, *P ≤ .05, *** *P* ≤ .001). Below, Western blot analysis was used to determine SB3 in +rhSB3 and MON^SB3+^ condition for both HUCCT1 and SG231 cells. (B) Invasion assay using Matrigel‐coated transwells. Cells counted and normalized to migrated MON or MON^CTR^ respectively (n = 3). Mean ±SEM (*P* value vs MON or MON ^CTR^ by Student's *t* test, *P ≤ .05, **P ≤ .01). (C) Cell proliferation analysis evaluated by BrdU incorporation using a colorimetric immunoassay. Absorbance values were normalized to MON or MON^CTR^ respectively (n = 3). Mean ± SEM (*P* value vs MON or MON ^CTR^ by Student's *t* test, *P ≤ .05, **P ≤ .01, ***P ≤ .001). (D) SB3 paracrine and endogenous effect on CCA‐SPH volume. Mean ± SEM (*P* value vs MON or MON^CTR^ by Student's *t* test, *P ≤ .05, **P ≤ .01, ***P ≤ .001). (E) Representative images of CCA SPH are shown (original magnification 40×, scale bar 50 μmol/L)

As expected, transfected cells showed a dramatic SB3 increase at mRNA level compared to the expression in SPH cells (Figure 2A). Interestingly, the exogenously added SB3 did result in a significant change in protein content for this factor, beside no modulation of its mRNA levels (Figure 2A, Figure S3). A similar discrepancy is shown in Figure 1.

We next evaluated the role of SB3 in the acquisition of stem‐like functional properties (sphere forming and invasion ability) as well as proliferation ability in both our models (Figure 2B‐E, Figures S3 and S4).

The in vitro invasive properties in the endogenous (MON^SB3+^) and the paracrine (+rhSB3) models were significantly increased for all tested CCA cell lines (Figure 2B, Figure S3 and S4). Importantly, this correlated with cellular phenotype modifications as shown by the elongated morphology highlighted by cristal violet staining and the enhanced amount of actin filament after phalloidin immunofluorescence (Figures S3 and S4).

Notably, SB3 impacted the proliferative capacity assessed by BrDU assay, especially in the MON^SB3+^ condition in both CCA cell lines (Figure 2C).

More importantly, regardless of the used in vitro approach, SB3 highly affected sphere size rather than the spherical frequency (Figure 2D,E, Figures S3 and S4) thus suggesting a main role in the maintenance of stemness features.

Taken together, these data indicate that SB3 induces an increase in sphere size and is able to sustain a highly invasive and proliferative phenotype of CCA cells.

**FIGURE 3:**
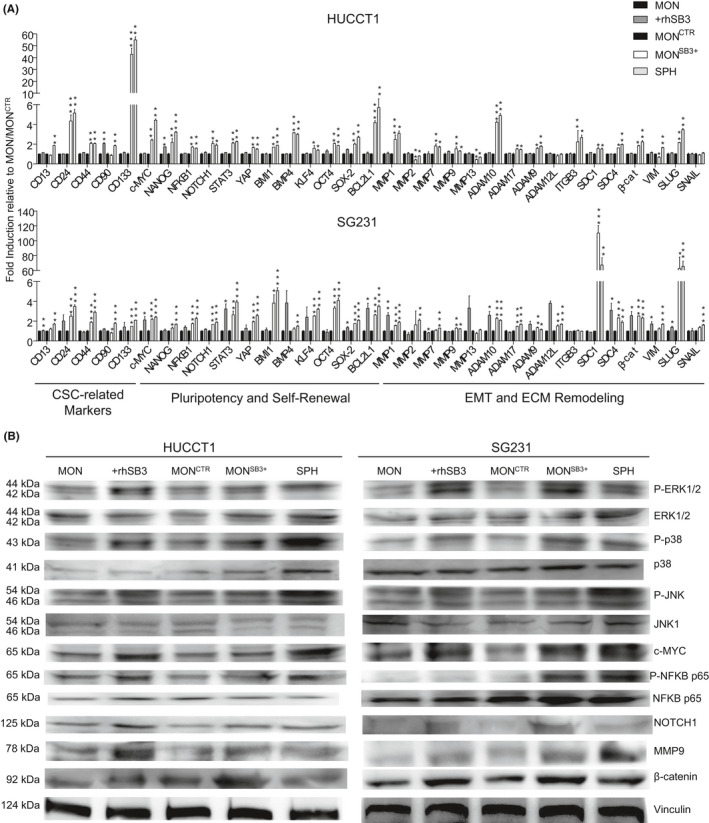
(A) Relative expression of transcript‐encoding liver CSC‐like, Self‐Renewal, Pluripotency, Epithelial Mesenchymal Transition (EMT) as well as Extracellular Matrix (ECM) remodelling genes in SB3 pretreated (rhSB3) or transfected (MON^+SB3^) of both HUCCT1 and SG231 cells. GAPDH was used as an internal control. Not treated monolayer (MON), monolayer transfected with control vector (MON^CTR^) as control. Sphere (SPH) condition was also evaluated. All mRNA levels are presented as fold changes normalized to 1 (mean expression of MON or MON^CTR^ respectively). Data are expressed as mean ± SEM (*P* value vs MON or MON^CTR^, respectively, by Student *t* test, *P ≤ .05, **P ≤ .01***P ≤ .001). (B) Immunoblot analysis in SB3 monolayer (MON^SB3+^) and control vector (MON^CTR^) in both transfected HUCCT1 and SG231 cells. Sphere (SPH) condition was also tested. Phospho ERK1/2, total ERK1, phospho p38, total p38, phospho JNK, total JNK1, c‐MYC, phospho NFKB p65, total NFKB p65, NOTCH1, MMP9 and β‐catenin. Vinculin immunoblot was performed to ensure equal loading

Analysis of the mRNA expression of stem‐like (CD24, CD44, CD90, CD133, c‐MYC, NOTCH1, STAT3, YAP, BMI1, OCT4, SOX2), EMT (β‐catenin, SLUG, SNAIL) and extracellular membrane (ECM) remodelling‐related genes (in particular MMP1, MMP7, MMP9, ADAM9, ADAM10, ADAM17, ITGB3) showed a marked and significant upregulation in both HUCCT1 and SG231 MON^SB3+^ cells, in comparison to mock‐transfected controls (MON^CTR^) (Figure 3A). Of note, the same genes were not affected by exposure of MON to exogenous rhSB3 (Figure 3A, Figure S3). Changes in gene expression were also associated with activation of key molecular pathways such mitogen‐activated protein kinases (ERK1/2, p38, JNK1), phosphorylation of NFκB (p65) as well as up‐regulation of c‐MYC, NOTCH, MMP9 and β‐catenin compared to MON^CTR^ cells in both HUCCT1 and SG231 cells (Figure 3B).

### 2.2 In vivo tumorigenicity of SB3

**FIGURE 4:**
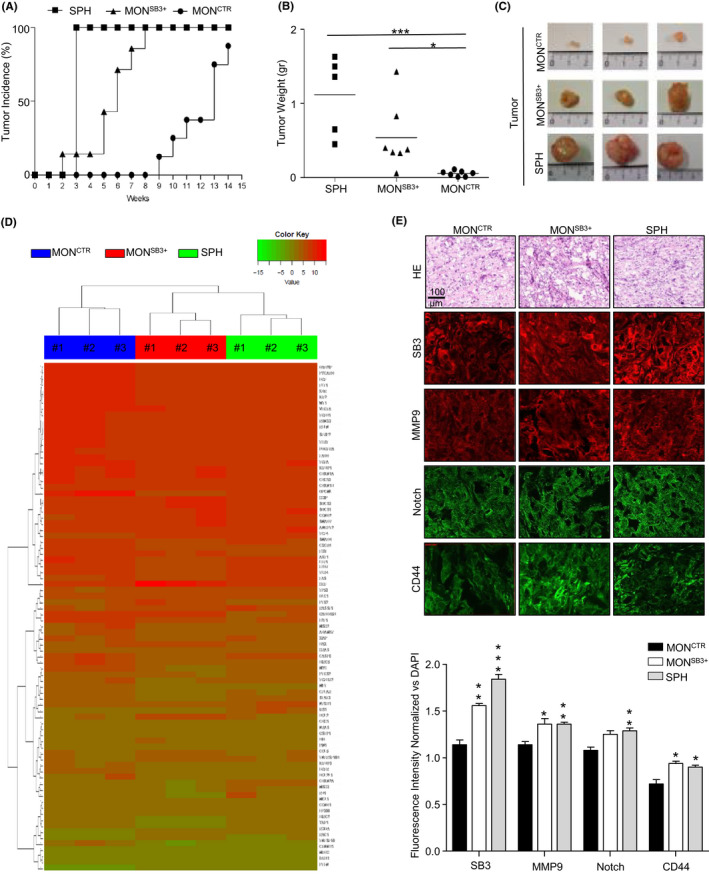
SB3 tumorigenic capacity in NSG mice. A) Tumour growth kinetic (n = 10), (B) weight of generated tumours at 15 weeks after subcutaneous injection into NSG mice of 1000 SPH, MON^SB3+^ and MON^CTR^ isolated cells as monitored by weekly palpation. Mean ± SEM (*P* value vs MON^CTR^ by Student's *t* test, * *P* ≤ .05, *** *P* ≤ .001). (C) Representative images of SPH, MON^SB3+^ and MON^CTR^‐derived tumours. (D) qRT‐PCR arrays focused on liver cancer pathways. Heatmap of different tumour samples based on the expression of 84 genes. Gene expression levels are expressed in colour code from green (low) to red (high) according to the colour key scale bar. Hierarchical clustering was based on complete linkage on Euclidean distances between genes (rows) or samples (columns). (E) Representative images of haematoxylin and eosin staining and of immunofluorescence for SB3 (red), MMP9 (red), CD44 (green) and NOTCH (green) on MON^CTR^, SPH, MON^SB3+^‐derived tumours in NSG mice. Original magnifications 63×. Below, quantification of the fluorescence of the analysed proteins by Axiovision software. Data were expressed as the mean of fluorescence of each protein vs Dapi measurement for each acquisition ±SEM. Statistical significance was assessed by Student t test (*P ≤ .05, **P ≤ .01***P ≤ .001)

Based on the above *in* vitro findings, for in vivo experiments, we decided to focus on the endogenous model of SB3 overexpression via transfection. We compared tumour formation and weight after injection of MON^SB3+^ or MON^CTR^ HUCCT1 cells into immune‐deficient NOD‐SCID/IL2Rgnull (NSG) mice (Figure 4).^10^ SPH were used as a positive control. Consistently with their greater in vitro self‐renewal ability, MON^SB3+^ were highly tumorigenic and engrafted earlier compared to MON^CTR^ (Figure 4A). Moreover, MON^SB3+^‐derived tumours had significantly higher weight and volume compared to MON^CTR^‐derived tumours (Figure 4B,C, Figure S5). Based on the results described above, we next performed a molecular characterization of tumour tissues. Gene expression profiles derived from a PCR array specific for liver cancer pathways revealed common upregulated genes in tumours from mice injected with SPH and MON^SB3+^ cells (Figure 4D). These included markers of extracellular matrix and cell adhesion molecules (ie ADAM17, CDH1 (E‐Cadherin), CDH13, CFLAR, EP300, ITGB1, PYCARD, RELN), EMT (ie β‐catenin, LEF1, TGFB1) as well as genes involved in signal transduction such as the classical Wnt pathway (ie CCND1, β‐catenin, FZD7, LEF1, MTDH, SFRP2, TCF4), TGFβ signalling (ie TGFB1, TGFBR2), IGF / IGFR signalling (ie IGF2, IGFBP1, IGFBP3), Hippo signalling (ie YAP1) and EGFR signalling (ie EGF, EGFR, TGFA). Overall, the over‐expression of genes involved in extracellular matrix degradation, EMT and TGFβ pathways as well as the involvement of those linked to Wnt and Hippo signalling, clearly confirmed the in vitro molecular and functional data (Figures 2 and 3).

Of note, gene cluster analysis showed that tumours from mice injected with SPH and MON^SB3+^ were more similar than those derived from MON^CTR^ (Figure 4D).

In agreement with the in vitro findings, SB3 overexpression in MON^SB3+^ and SPH‐derived tumours was associated with an increase in NOTCH, MMP9 and the CSC‐related marker CD44 expression, at the protein level (Figure 4E).

Overall, although slightly smaller in size, tumours generated by MON^SB3+^ cells were very similar in terms of incidence and molecular characterization to those generated by SPH cells, suggesting SB3 as an excellent modulator of tumorigenesis in CCA.

### 2.3 The CCA stem‐like subset is sensitive to SB3 interference

**FIGURE 5:**
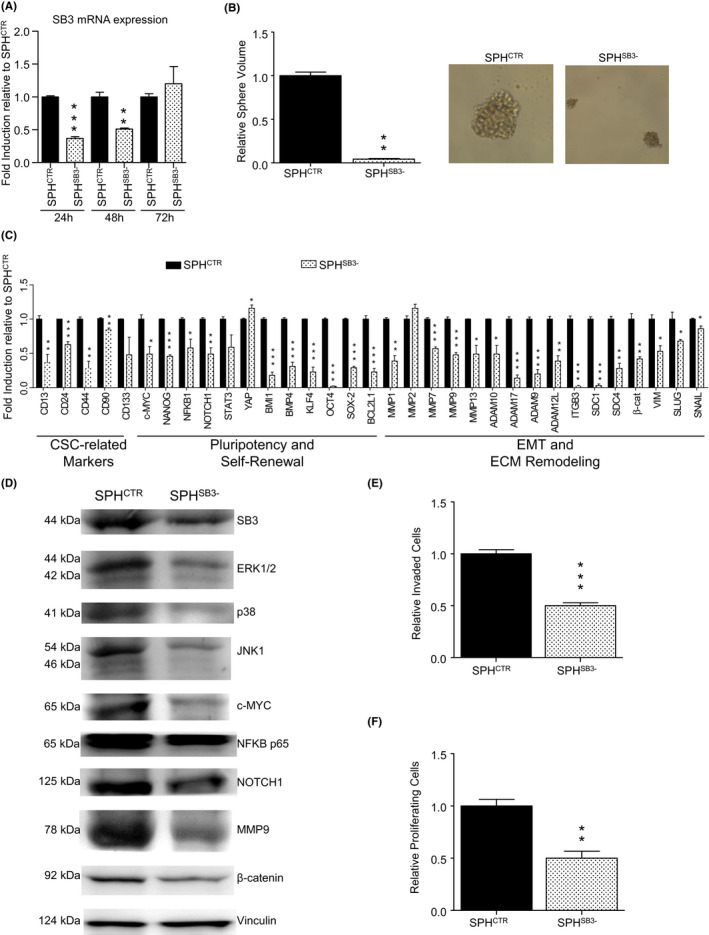
Effects of SerpinB3 silencing in CCA SPH cells (SPH^SB3‐^). (A) SerpinB3 (SB3) gene expression levels following siRNA transfection, presented as fold changes normalized to mean expression of siCTR SPH (SPH^CTR^). mRNA levels at 24, 48 and 72 hours after transfection (n = 4), Mean ±SEM (*P* value vs SPH^CTR^ by Student's *t* test, **P ≤ .01, ***P ≤. 001 vs S). (B) Effects of SB3 silencing on CCA sphere‐volume (n = 3). Mean ± SEM (*P* value vs SPH^CTR^ by Student's *t* test, **P ≤ .01). Representative images of CCA SPH are shown (original magnification 40×, scale bar 50 μmol/L). (C) Expression of different genes, expressed as fold changes normalized to mean expression of SPH^CTR^ sample (n = 3). Mean ± SEM (*P* value vs SPH^CTR^ by Student's *t* test, *P ≤ .05, **P ≤ .01, ***P ≤ .01). Gene groups are indicated at the bottom of the barograms. (D) Immunoblot of different proteins following SB3 silencing. (E) Invasion of SB3‐silenced HUCCT1 SPH was measured in modified Boyden chambers (n = 5). Mean ± SEM (*P* value vs SPH^CTR^ by Student's *t* test, ***P ≤ .001). (F) Cell proliferation analysis evaluated by BrdU incorporation using a colorimetric immunoassay. Absorbance values were normalized to SPH^CTR^ (n = 3). Mean ± SEM (*P* value vs SPH^CTR^ by Student's *t* test, **P ≤ .01)

Due to the high SB3 levels in CCA SPH, we tested the effects of genetic knockdown of this molecule on functional and molecular features. As expected, SB3 depletion of HUCCT1 SPH (Figure 5A) markedly reduced CCA stem‐like properties such as in vitro spherogenicity (Figure 5B, Figure S6) and expression of stem‐like genes (Figure 5C). These functional effects were accompanied by a marked downregulation of signalling pathways including mitogen‐activated protein kinases (ERK1/2, p38, JNK1), NFκB (p65) as well as c‐MYC, NOTCH, MMP9 and β‐catenin (Figure 5D) that were clearly enhanced after SB3 exogenous or endogenous stimulation (Figure 3).

Similarly, SB3 silencing significantly reduced the ability to invade a basement‐membrane‐like matrix (Figure 5E, Figure S6) and cell proliferation (Figure 5F), major properties of highly malignant tumour cells. Overall, these data suggested SB3 as a critical regulator of stemness and an innovative therapeutic candidate in CCA.

### 2.4 Expression of SB3 is associated with a more aggressive course in CCA patients

**FIGURE 6:**
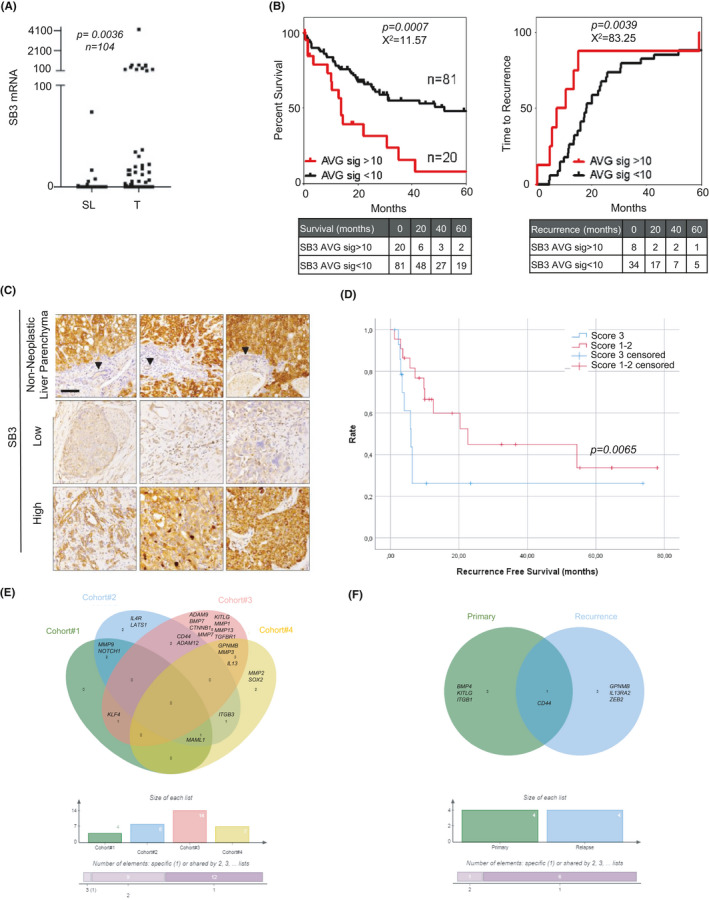
(A) Scatter dot plot of 104 tumour samples (T) vs 59 matched surrounding livers (SL) showing a significantly higher expression of SB3 mRNA in the tumours. Mann‐Whitney test (2‐tailed) was used for significance. (B) Overall survival (OS, 5 years) (left) and time to recurrence (months over 5 years) (right) for 104 CCA patients sub‐grouped based on SB3 expression. Patients were stratified with an average signal of 10 as the cut off value. Samples <10 would be ‘no/low expression’ and samples >10 would be ‘high’. With that stratification the association of SB3 mRNA expression with survival and time to recurrence was analysed Log‐rank and Gehan‐Breslow‐Wilcoxon KM curves respectively. (C) Representative SB3 low‐ and high‐grade immunohistochemical staining in the considered CCA series (n = 38). Normal bile ducts of non‐neoplastic liver parenchyma are indicated by the black arrows. (Original magnifications 20×; magnification bar = 100 µm). (D) Kaplan‐Meier of the time to recurrence (months) in CCA cases divided according to the SB3 score determined by immunohistochemistry (n = 38) (high SB3 = score 3, low SB3 = score 1‐2). Survival Curves were estimated using Kaplan‐Meier method and the differences between curves was assessed by long‐rank test. (E) Venn diagram showing the number and overlap of genes significantly positively correlated with SERPINB3 in four different CCA cohorts (Cohort#1=GSE26566, Cohort#2=GSE45001, Cohort#3=EGA00001000950, Cohort#4=TCGA database) (F) Venn diagram showing the number and overlap of genes significantly positively correlated with SERPINB3 in 10 primary iCCA tumours and 10 iCCA recurrent tumours (data from dataset GSE107102)

To explore the possible clinical relevance of our in vitro and in vivo data, we analysed SB3 expression and its relation to pathological features and clinical outcomes in human iCCA specimens. In order to evaluate the potential clinical relevance in terms of CCA development and prognosis, SB3 mRNA presence was investigated in a published dataset of iCCA patients (n = 104).^29^ On average, expression of SB3 mRNA was higher in tumours than in matched surrounding liver (Figure 6A). In addition, 20 out of 104 cases of iCCA showed high SB3 expression (average intensity signal of 10 as cut‐off value; high expression >10; lower/no expression <10). Remarkably, patients with high SB3 expression had a significantly lower survival (Log‐rank curve) and shorter time to recurrence (Gehan‐Breslow‐Wilcoxon curve) than those with low SB3 (Figure 6B). This datum was further validated in the Nakamura cohort^30^ (Figure S7).

Next, SB3 expression at the protein level was investigated by immunohistochemistry in a total of 38 formalin‐fixed, paraffin‐embedded human iCCA tissue specimens. collected partly in the tissue bank at the French liver biobanks network^31^, ^32^, ^33^ (n = 21) and partly in the tissue bank at Humanitas Clinical Institute (Rozzano, Italy) (n = 17). Variable degrees of SB3 expression were observed, as reported in Table 1 and exemplified in Figure 6C. Notably, patients with SB3 high scores had a three‐fold lower time to recurrence compared to patients with low SB3 expression (Figure 6D). Moreover, normal bile ducts did not express SB3 (Figure 6C).

**TABLE 1:** Clinical and pathological parameters of CCA patients used for immunohistochemical staining, with relative SerpinB3 expression scores

Patient ID	Cirrhosis	Tumor size (cm)	Necrosis	Perineural invasion	Recurrence	Recurrence‐free survival (months)	Overall survival (months)	SB3 score
#1	Yes	10	No	No	Yes	1.2	1.4	2
#2	No	6.3	Yes	No	No	32.4	31.9	1
#3	Yes	8	No	No	no	8.4	8.3	2
#4	No	17	Yes	No	Yes	6.3	20.8	3
#5	Yes	8	No	No	No	4.0	4.0	2
#6	No	8.5	Yes	No	Yes	3.0	5.3	2
#7	Yes	4.5	No	No	No	73.7	72.5	3
#8	Yes	11	No	No	Yes	12.5	38.3	2
#9	No	4	Yes	No	No	23.4	49.5	3
#10	No	5	No	No	Yes	54.4	97.0	2
#11	No	7.5	Yes	No	No	36.5	35.9	2
#12	No	3.5	Yes	Yes	No	77.9	76.6	2
#13	No	7	Yes	No	No	64.6	63.6	2
#14	No	5.5	No	Yes	Yes	6.3	13.3	2
#15	No	8	<10%	NA	Yes	3.6	8.7	2
#16	No	4	No	Yes	No	10.5	10.3	3
#17	Yes	5	Yes	No	No	11.3	11.1	2
#18	Yes	10.5	Yes	No	Yes	5.8	12.4	3
#19	No	5.6	No	Yes	Yes	7.2	19.2	1
#20	No	8	Yes	Yes	No	3.4	3.3	3
#21	No	5.5	No	No	NA	NA	0.1	2
#22	No	45	Yes	Yes	No	10.0	14.2	1
#23	Yes	28	No	No	No	18.0	25.3	1
#24	No	82	No	No	No	12.0	16.8	1
#25	No	59	Yes	No	Yes	3.0	5.4	3
#26	No	50	No	No	Yes	6.0	7.6	3
#27	No	30	No	Yes	Yes	4.0	4.9	3
#28	No	45	Yes	Yes	Yes	2.3	10.73	3
#29	No	30	No	Yes	No	55.33	55.33	1
#30	No	190	Yes	No	No	3.2	62.33	3
#31	No	25	No	Yes	Yes	22.6	7.6	2
#32	No	85	No	No	Yes	3.4	25.97	3
#33	No	100	Yes	Yes	Yes	20.3	7.47	2
#34	No	30	No	No	No	1.3	1.3	3
#35	No	40	No	No	Yes	9.97	28.17	1
#36	No	87	Yes	No	Yes	5.67	5.83	2
#37	Yes	60	No	No	Yes	2.77	3.03	3
#38	No	59	Yes	Yes	Yes	9.77	17.93	1

Additionally, in agreement with in vitro gene expression (Figure 3A), correlation analysis revealed that in the GSE26566^29^ and GSE45001^33^ database, SB3 mRNA correlated with MMP9 and NOTCH1 expression (Table 2). Notably, SB3 correlations with stem‐like genes were further validated in two additional public databases (Figure 6E, Table 2). Although only few genes were commonly correlated among the four databases, the association of SB3 with numerous stem‐related genes was evident.

**TABLE 2:** Correlation of SERPINB3 with a panel of selected genes in four different CCA cohort (Cohort#1 = GSE26566, Cohort#2 = GSE45001, Cohort#3 = EGA00001000950, Cohort#4 = TCGA database) using data from public database. Pearson correlation between gene pairs was calculated using R and the "cortest" function, yielding correlation coefficients and *P*‐values (in italic). In bold genes significantly positively correlated with SERPINB3 in each cohort

	Gene	Cohort#1		Cohort#2		Cohort#3		Cohort#4	
		R	*P* value	R	*P* value	R	*P* value	R	*p value*
Stem‐related Genes	BMP4	0.288	*0*.*217*	−0.027	*0*.*871*	0.086	*0*.*350*	0.225	*0*.*158*
	BMP7	0.108	*0*.*273*	0.152	*0*.*357*	**0**.**235**	***0*** . ***010*** **	0.225	*0*.*157*
	CD44	0.295	*0*.*182*	**0**.**425**	***0*** . ***007*** **	**0**.**507**	***0*** . ***000*** ***	−0.062	*0*.*700*
	KITLG	0.068	*0*.*810*	−0.371	*0*.*020* *	**0**.**406**	***0*** . ***000*** ***	0.129	*0*.*420*
	KLF4	**0**.**201**	***0*** . ***041*** *	0.273	*0*.*093*	**0**.**395**	***0*** . ***000*** ***	0.132	*0*.*409*
	LATS1	0.135	*0*.*486*	**0**.**521**	***0*** . ***001*** ***	0.171	*0*.*063*	−0.160	*0*.*317*
	LEF1	0.224	*0*.*271*	−0.460	*0*.*003* **	0.022	*0*.*809*	0.230	*0*.*147*
	MAML1	**0**.**402**	***0*** . ***030*** *	**0**.**493**	***0*** . ***001*** ***	−0.004	*0*.*964*	**0**.**323**	***0*** . ***039*** *
	NOTCH1	**0**.**528**	***0*** . ***003*** **	**0**.**437**	***0*** . ***006*** **	0.011	*0*.*905*	−0.067	*0*.*677*
	POU5F1	0.311	*0*.*101*	0.266	*0*.*102*	0.041	*0*.*656*	0.228	*0*.*152*
	SOX2	0.186	*0*.*334*	0.181	*0*.*271*	0.112	*0*.*226*	**0**.**542**	***0*** . ***000*** ***
	TGFBR1	0.489	*0*.*007* **	0.195	*0*.*234*	**0**.**189**	***0*** . ***039*** *	0.185	*0*.*246*
EMT and ECM Remodeling‐related Genes	ADAM10	0.328	*0*.*082*	−0.1889	*0*.*2495*	0.086	*0*.*353*	0.277	*0*.*080*
	ADAM12	—	—	**0**.**427**	***0*** . ***007*** **	**0**.**327**	***0*** . ***000*** ***	−0.141	*0*.*379*
	ADAM17	0.107	*0*.*579*	−0.224	*0*.*171*	0.140	*0*.*128*	0.019	*0*.*906*
	ADAM9	—	—	−0.309	*0*.*056*	**0**.**235**	***0*** . ***010*** **	0.056	*0*.*730*
	CTNNB1	0.150	*0*.*129*	−0.222	*0*.*174*	**0**.**416**	***0*** . ***000*** ***	0.131	*0*.*415*
	GPNMB	0.115	*0*.*551*	−0.182	*0*.*269*	**0**.**185**	***0*** . ***044*** *	**0**.**313**	***0*** . ***046*** *
	IL13	0.080	*0*.*422*	—	—	**0**.**205**	***0*** . ***025*** *	**0**.**327**	***0*** . ***037*** *
	IL4R	0.097	*0*.*615*	**0**.**548**	***0*** . ***000*** ***	0.012	*0*.*897*	0.141	*0*.*378*
	ITGB3	0.069	*0*.*721*	**0**.**376**	***0*** . ***018*** *	0.067	*0*.*470*	**0**.**329**	***0*** . ***036*** *
	MMP1	0.243	*0*.*263*	0.273	*0*.*093*	**0**.**437**	***0*** . ***000*** ***	0.307	*0*.*051*
	MMP13	0.261	*0*.*218*	—	—	**0**.**499**	***0*** . ***000*** ***	−0.075	*0*.*641*
	MMP2	0.133	*0*.*537*	−0.452	*0*.*004* **	0.031	*0*.*739*	**0**.**519**	***0*** . ***001*** ***
	MMP3	0.400	*0*.*065*	—	—	**0**.**487**	***0*** . ***000*** ***	**0**.**668**	***0*** . ***000*** ***
	MMP7	0.306	*0*.*107*	0.016	*0*.*925*	**0**.**240**	***0*** . ***009*** **	−0.089	*0*.*580*
	MMP9	**0**.**488**	***0*** . ***007*** **	**0**.**478**	***0*** . ***002*** **	0.027	*0*.*769*	0.014	*0*.*931*
	SDC1	0.022	*0*.*825*	−0.061	*0*.*712*	0.033	*0*.*722*	0.066	*0*.*684*
	SNAI1	0.059	*0*.*761*	−0.295	*0*.*068*	0.142	*0*.*124*	0.211	*0*.*184*

* *P* ≤ .05

** *P* ≤ .01

*** *P* ≤ .001.

The lack of commonly correlated genes could be due to differences in the molecular transcriptomic analysis performed (ie different probes), or possibly in the staging and number of CCA tissues analysed (Figure 6E, Table 2). In this regard, the correlative analysis in a small cohort (GSE107102^34^) including iCCA (n = 10) and recurrent iCCA (n = 10) suggested that SB3‐associated genes might change along with the disease progression (Figure 6F, Table S1).

These results indicate that in patients with iCCA, higher SB3 expression correlates with expression of genes involved in tumour progression and is associated with significantly worse clinical outcomes.

## 3 DISCUSSION

Clinical and translational data obtained in recent years have led to an improvement in the treatment of CCA. Nonetheless, recurrence and mortality rates remain unacceptably high in this tumour type, making the identification of novel pathways implicated in CCA development or progression an urgent priority. Accumulation of CSCs and dysregulation of stemness‐associated pathways are important contributors to CCA resistance, recurrence and metastasis. Therefore, a deeper understanding of the CSC molecular features may be of great significance for the CCA treatment.

The findings reported in this study identify SB3 as a novel and pivotal modulator of the stemness features of CCA. These conclusions are supported by different and integrated lines of evidence, encompassing in vitro experiments, in vivo tumour xenograft models, and correlation of SB3 expression with clinical outcomes in patients.

Experiments in cultured cells indicated that SB3 expression was markedly up‐regulated in SPH stem‐like cells, consistent with their high in vivo tumorigenic potential and superior stemness features.^10^ At a functional level, both SB3 overexpressing or treated CCA cells had larger spheres and higher invasive capacity. Regarding the size of spheres, it is generally believed that stem cells give rise to larger spheres while progenitor cells which lose the ability of self‐renewal, to smaller ones. The sphere size obviously reflects the proliferation of sphere‐forming cells.

The modifications in the functional properties were associated with relevant alterations in a number of signalling pathways involved in cancer development and progression. Specifically, a direct relation between SB3 expression and activation of members of the mitogen‐activated protein kinase family ERK, p38 and JNK was demonstrated.

It is well known that MAPKs coordinately regulate diverse cellular activities including mitogenesis, cell motility, survival and apoptosis. In particular, it is commonly accepted that activated ERK1 and ERK2 phosphorylate numerous molecules, including nuclear substrates such as c‐MYC, and STAT3 that were significantly overexpressed at mRNA level in MON^SB3+^ cells. Similarly, it is well known that activated p38 phosphorylates several transcription factors including NFκB, whose phosphorylation was increased in SB3‐transfected CCA cells. NFκB activation plays critical roles in many of the ‘hallmarks’ of cancer, such as tumour cell proliferation, suppression of apoptosis, activation of angiogenesis and EMT induction. As expected, our in vitro findings were confirmed in vivo, using a subcutaneous CCA mouse model in immunocompromised NSG mice. Of note, tumours derived by MON^+SB3^ cells faithfully reproduced SPH‐derived tumour incidence and molecular features, thus indicating SB3 as a strategic factor for in vivo self‐renewing CCA cells.

Although SB3 was reported to be upregulated in many poorly differentiated and advanced metastatic tumours, there is only partial evidence of its functional contribution to malignancy.^35^, ^36^ However, to date, no studies have described the role of SB3 in CCA. In liver cancer cells, SB3 acts as an autocrine and/or paracrine mediator, inducing apoptosis resistance,^37^ cell proliferation,^38^ EMT and increased invasiveness^18^, ^19^ by NFκB activation as well as c‐Myc expression through the Yap pathway.^22^ Notably, the involvement of SB3 has been described as a relatively early event in hepatocarcinogenesis,^24^ in embryonic liver tumours (hepatoblastoma)^28^ as well as in liver stem/progenitor cells positive for the hepatic EpCAM, both in human foetal livers and in adult livers with cirrhosis.^39^ Furthermore, liver tumours with stemness signature are highly aggressive, and along this line, the highest levels of SB3 are overexpressed, in the subset of aggressive forms of HCC, characterized by early tumour recurrence after surgical resection.^20^ Overall, the SB3 mechanisms of action are still poorly understood. However, evidence indicated that SB3 can induce constitutive activation of the unfolded protein response (UPR) which represents a well‐known cellular stress‐response pathway.^40^ UPR is an adaptive cellular program to cope with protein misfolding stress and to adapt the endoplasmic reticulum folding capacity. During tumour development, cancer cells are facing intrinsic (an acute demand of protein synthesis to support tumour proliferation, migration and differentiation, after oncogenic activation) and extrinsic (limiting nutrient or oxygen supply) challenges.

SB3 is responsible for UPR activation, possibly via its inhibition of the lysosomal proteases.^40^ Because of elevated UPR, SB3 induces NF‐κB and the expression of pro‐inflammatory cytokines, predominantly IL‐6, which leads to an EMT‐like phenotypical change and oncogenic transformation in the mammary epithelial MCF10A cells.^40^

Importantly, there are clearly strong links between the UPR and MAPK signalling networks.^40^ Obviously, based on this evidence an in‐depth study of UPR signalling in CCA cells should be performed in order to explain the in vitro and in vivo effects on invasion and self‐renewal in the near future.

Furthermore, from the point of view of the controlling mechanisms of SB3 expression, our findings indicated a clear discrepancy between mRNA and protein expression that could be attributed to a post‐transcriptional SB3 regulation.

An intriguing finding in our study is that MON^+SB3^ cells express higher levels of MMP1, MMP7 and MMP9 and of several members of the ADAM family. Overexpression of SB3 also up‐regulated the β3 integrin chain, which regulates cancer cell survival, tumour initiation and tumour stemness.^41^, ^42^ Changes in the regulation of matrix‐remodelling genes could be the molecular basis of the observed increase in the invasive potential of SB3‐transfected cells, another feature of the aggressive phenotype of CCA. It is important to underscore that we found a strong, direct correlation between the expression of SB3 and of MMP9 in a dataset of patients with iCCA. Disease recurrence and metastasis are the primary cause of cancer treatment failure and patient death. Proteolytic degradation of the ECM is considered an essential step in the invasion and metastasis of malignant cells to distant tissues, and proteases, such as MMPs, expressed by neoplastic and/or stromal cells are considered key players in this process.

A very relevant result of this study is the identification of SB3 as a biomarker which identifies patients with poor prognoses. SB3 mRNA levels were higher in CCA than in surrounding liver tissue when a dataset of patients was analysed. More importantly, in CCA patients with high expression levels of SB3, overall survival and time to recurrence were significantly shorter than in patients with low SB3. Because mRNA may not be routinely available after surgical resection in common practice, we also analysed the expression of SB3 using immunohistochemistry in another set of patients. Strikingly, also in this case patients with high SB3 signal had a markedly shorter time to recurrence than those with low expression levels. These data underscore the role of SB3 not only as a potential target for blocking the progression of CCA, but also as a means to identify those patients with a higher likelihood to have a negative outcome.

It is worth emphasizing that we applied two different in vitro experimental approaches: endogenous SB3 as well as the paracrine effect of the exogenously added molecule. In particular, the choice to test SB3 effects according to the exogenous modality is mainly due to the typical feature of CCA of being surrounded by a highly desmoplastic microenvironment. Although in the present study we mainly focused on the characterization of the intracellular effects of SB3, the external impact of this molecule deserves to be explored in detail in the near future. Nevertheless, the overall effect of the two approaches (endogenous and exogenous) on spherogenicity, invasion, proliferation as well as protein overexpression and activation was almost identical.

Despite ta little modulation at the mRNA level after exogenous treatment, the in vitro effects at functional and protein level clearly reflect those of transfected cells. The underlying mechanism needs to be investigated in detail.

In conclusion, our study provides evidence for SB3 as a new molecule involved in the biology of the CCA stem‐like compartment, possibly supporting tumour fate and CSC formation. Based on these results, interference with SB3‐generated signals should be further investigated for its potential therapeutic relevance, and SB3 expression in CCA deserves to be analysed as a prognostic biomarker in a larger series of patients.

## 4 MATERIAL AND METHODS

### 4.1 Analysis of SB3 protein level in CCA tissues

A total of 38 formalin‐fixed, paraffin‐embedded human iCCA specimens were analysed for the immunohistochemical expression of SB3. All specimens were obtained after surgical resection and collected partly in the tissue bank at the French liver biobanks network^31^, ^32^, ^33^ (n = 21) and partly in the tissue bank at Humanitas Clinical Institute (Rozzano, Italy) (n = 17) in accordance with informed consent retrieved from patients and local ethics committee approval.

The immunohistochemical expression of SB3 (polyclonal; rabbit; Hepa‐Ab, Xeptagen) was performed on the automated Leica Microsystems Bondmax® (Leica). This antibody recognizes both SerpinB3 and SerpinB4 isoforms. Both cytoplasmic and nuclear staining was retained for scoring. Immunostaining was semiquantified using a three‐tier scoring based on intensity of staining (1 = weak; 2 = moderate; 3 = strong). Correlation analysis with clinicopathological CCA data was conducted by GraphPad Prism v5.

### 4.2 Survival analysis

The Kaplan‐Meier estimator was used to establish the association of SB3 expression with overall survival. Median expression of SB3, respectively, was used to stratify patients in Nakamura^30^ cohorts. The expression above AVG Sig >10 was used to stratify Andersen cohort.^29^ Curves competence was performed with Log‐Rank test (GraphPad Prism 9.1.1).^29^

### 4.3 Correlation analysis

Gene expression profiles were obtained from the publicly available data: GSE26566,^29^ EGA00001000950^30^ and GSE45001^33^ and The Cancer Genome Atlas (TCGA) database.^43^

For the microarray gene expression data, the AVG signal/log2AVG signal was used for the correlation analysis and RPKM (Reads Per Kilobase Million) for 119 iCCA samples in the EGA00001000950 dataset. Spearman correlation was applied (GraphPad Prism 9.1.1) independently in each cohort.^29^, ^31^

For the analysis of primary and recurrent iCCA, the expression data (log intensity values) of a panel of stemness‐related genes was extrapolated from the external dataset GSE107102.^34^ This included the profile of 10 primary iCCA tumours (PR) and of 10 iCCA recurrent (REC) tumours (3 iCCA tissues and 7 ascites samples, isolated from ascites liquid). Pearson correlation between SB3 and the 38 stem‐enriched gene list was calculated in primitive and recurrent iCCA tumours separately. Genes with a Pearson coefficient greater than 0.5 or lower than −0.5 were selected as positively or negatively correlated respectively.

Venn diagrams created using JVenn software (http://jvenn.toulouse.inra.fr/).

### 4.4 In vivo xenograft model

NOD/SCID/IL2rγ null (NSG) mice (Jackson Laboratory) at 6 weeks of age were used in all experiments. In vivo experiments were performed in accordance with the guidelines and approval of the local Experimental Animal Committee. A quantity of 1,000 sphere‐ or monolayer‐derived SB3 transfected and control HUCCT1 cells were dissociated into single‐cell suspensions and resuspended in 100 μL of DMEM and reduced Matrigel growth factor (BD Bioscience) (1:1), and the mixture was s.c. injected into the right flank of 6‐week‐old NSG mice. Tumour growth was monitored, and the diameter of the growing tumours was measured in millimetres every week using a calliper. The animals were sacrificed when the xenografts reached 2.0 cm in diameter.^10^

### 4.5 Statistical analysis

Student's *t* test, one‐way ANOVA and Tukey post hoc test, two‐way ANOVA, Bonferroni post hoc test for comparison. Kaplan‐Meier graph and Log rank test for survival significance. GraphPad Prism v5 was used for data analysis. The error bars represent 1 ± SEM. The *P* value was calculated with Student's *t* test. The statistical significance and *P* value are shown when relevant.

Immunofluorescence, western blotting, quantitative reverse‐transcriptase polymerase chain reaction (qRT‐PCR), were performed using standard assays described in the Supporting Materials and Methods.

## ETHICAL APPROVAL STATEMENT

The study was performed in accordance with the guidelines of the Helsinki Declaration and approved by Humanitas Research Committee.

## CONFLICTS OF INTEREST

The authors declare no competing interests.

## AUTHORS’ CONTRIBUTIONS

CR, FM and PP designed the study and wrote the manuscript. MC, AC, MP, BP, GL, DVPNO, ML, PM‐G, JBA, CC, LS, CPN, GC, SQ, AB, MF, MR, MP, SR, LDT, CC, PI and GT provided materials, performed the experiments, collected the data and analysed the results. CR, FM and PP supervised the project and critically revised the manuscript. All authors have read and approved the final manuscript.

## PATIENT CONSENT STATEMENT

Fully informed consent was obtained from all patients.

## PERMISSION TO REPRODUCE MATERIAL FROM OTHER SOURCES

No materials reproduced from other sources in this manuscript.

## Supporting information

Fig S1‐S7

Table S1

Supporting Information

## Data Availability

The data that support the findings of this study are available from the corresponding author [CR], upon reasonable request.
