# Supplementary figures and images for: The protease‐inhibitor SerpinB3 as a critical modulator of the stem‐like subset in human cholangiocarcinoma
Source: Liver Int. 2021 Sep 16;42(1):233–48. doi: 10.1111/liv.15049 (PMC9290104; doi:10.1111/liv.15049)

Suppl. Fig.1

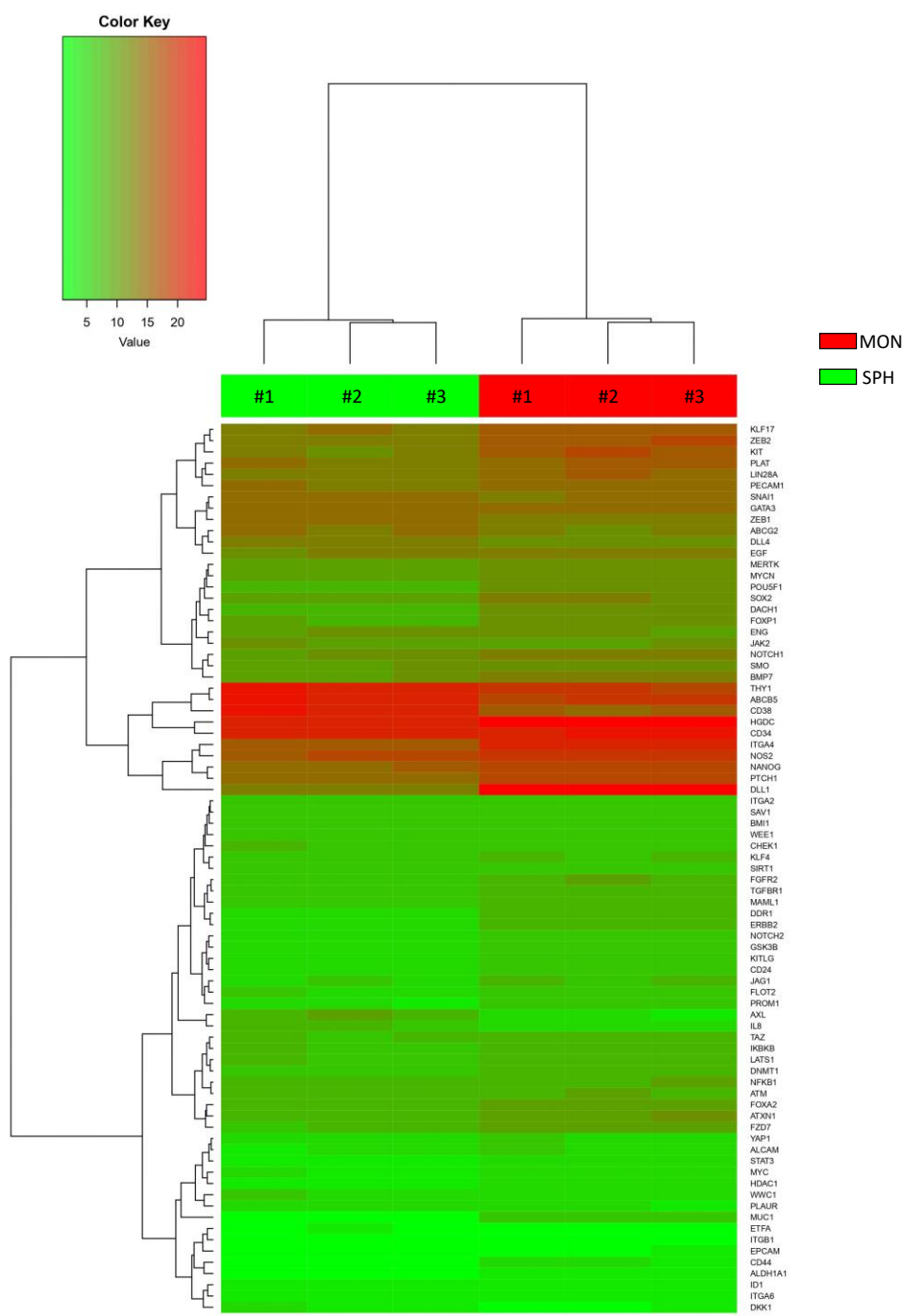

Suppl. Fig.2

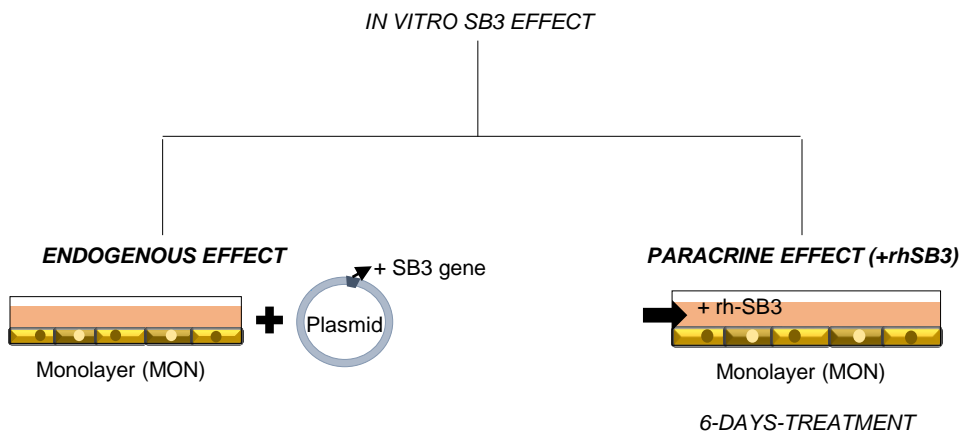

**A**

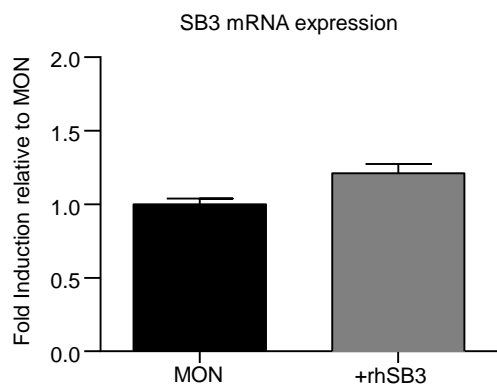

**B**

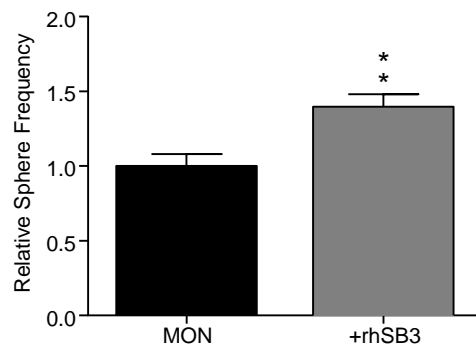

**C**

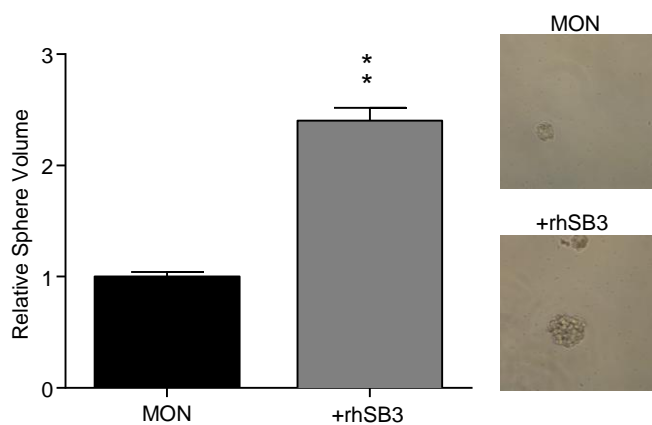

**D**

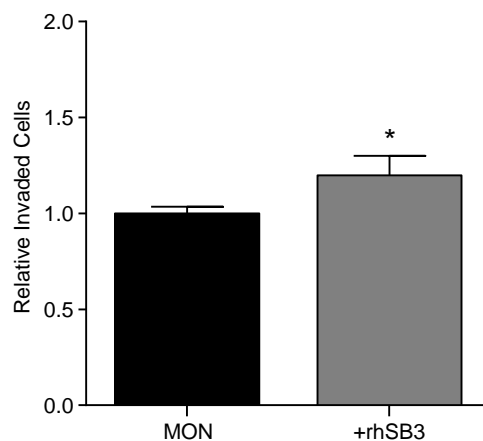

**E**

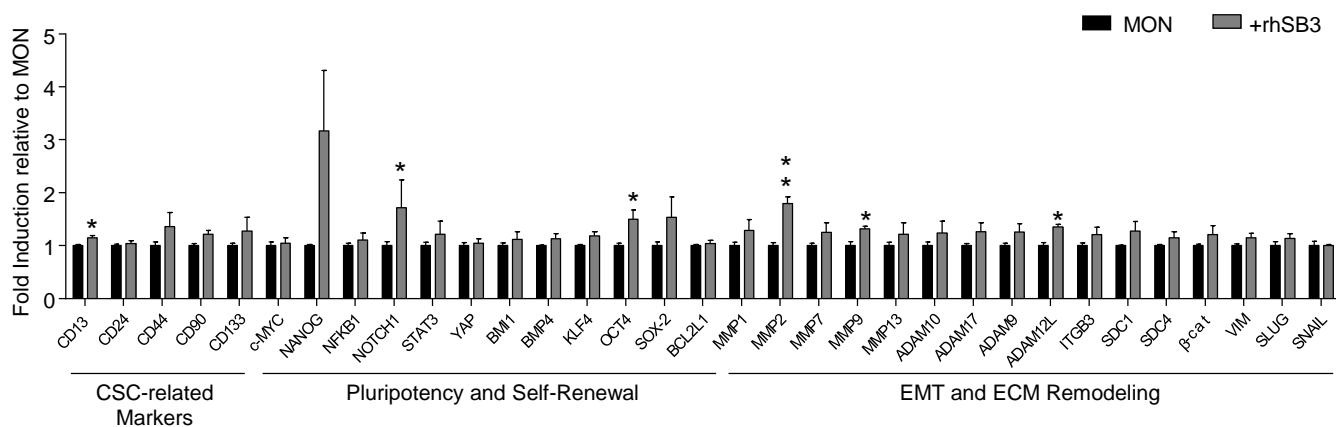

**F**

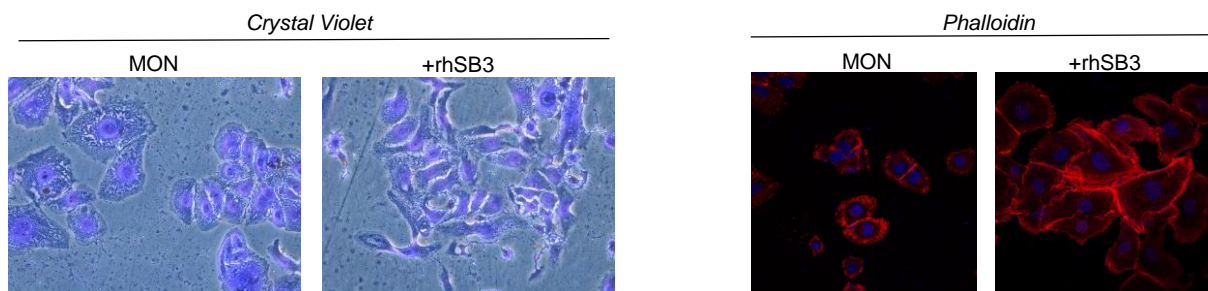

**A**

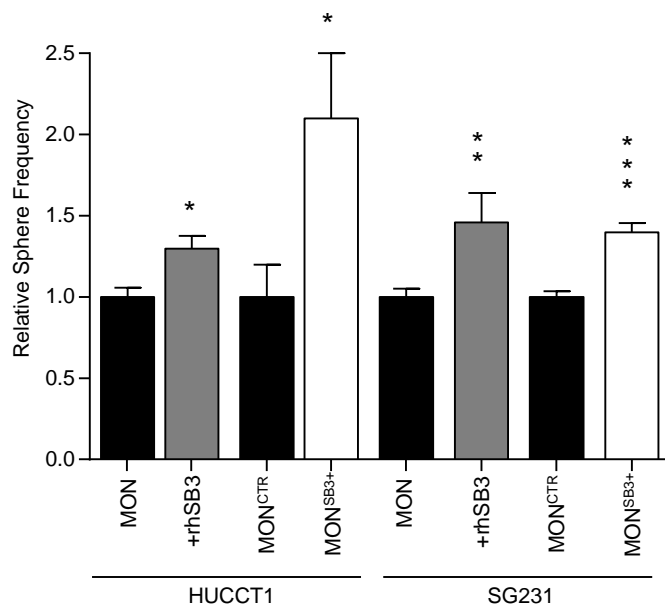

**B**

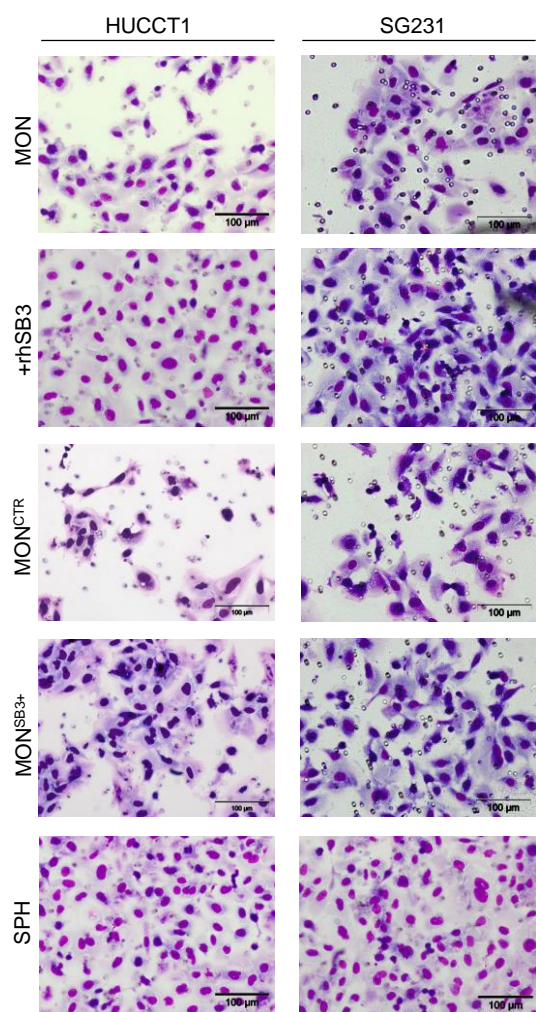

**C**

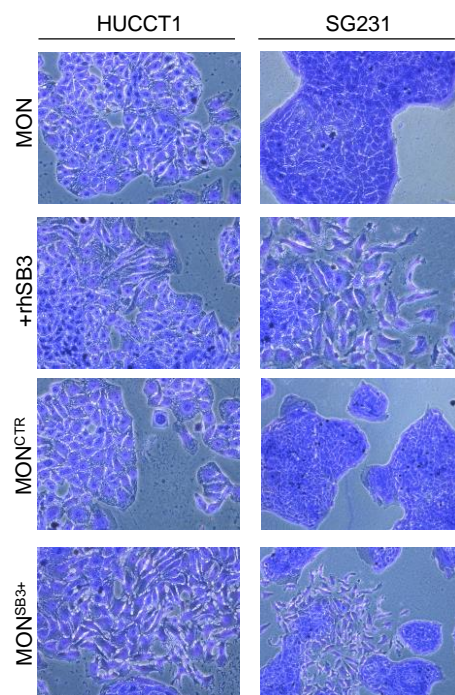

**D**

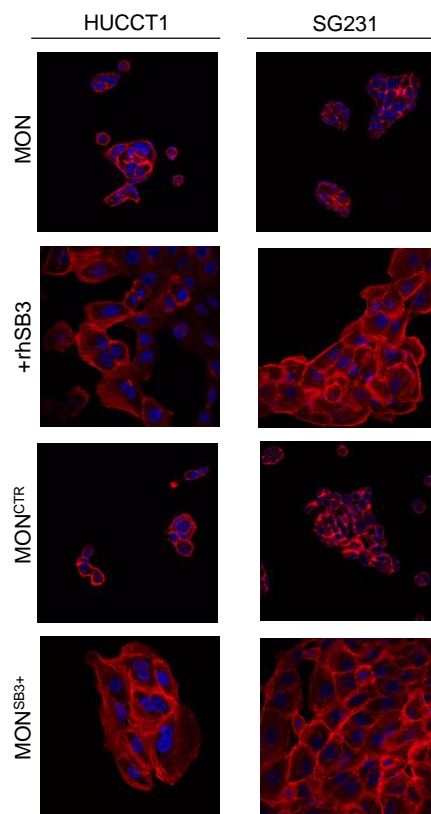

Suppl. Fig.5

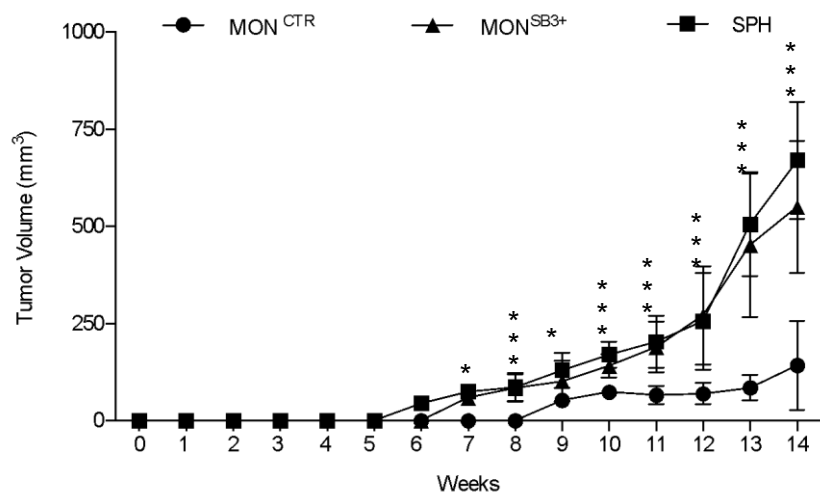

Suppl. Fig.6

A

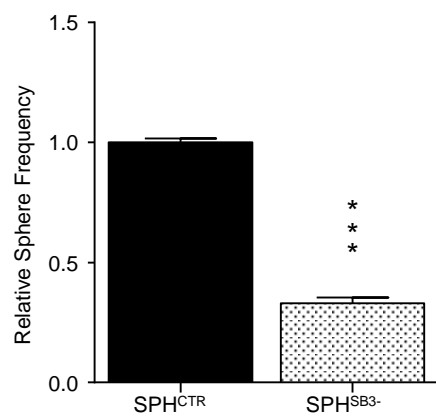

B

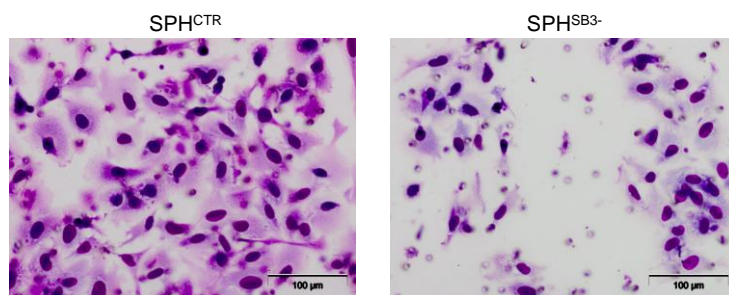

Suppl. Fig.7

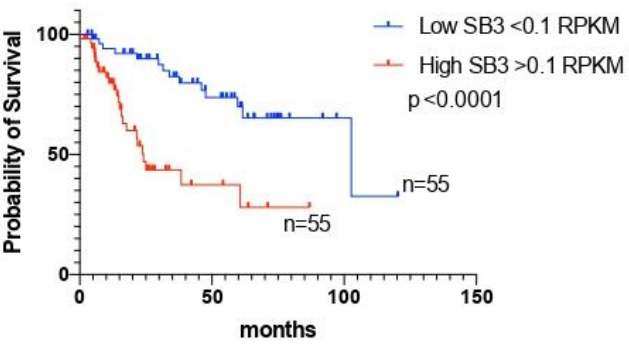

Supplement: Supplementary file 1 — Fig S1‐S7 [file LIV-42-233-s003.pdf]
