# Supplementary material for: The protease‐inhibitor SerpinB3 as a critical modulator of the stem‐like subset in human cholangiocarcinoma
Source: Liver Int. 2021 Sep 16;42(1):233–48. doi: 10.1111/liv.15049 (PMC9290104; doi:10.1111/liv.15049)
Supplement: Supplementary file 2 — Table S1 [file LIV-42-233-s001.pdf]

Suppl. Table 1

Correlation of SERPINB3 with a panel of selected genes in 10 primary iCCA tumors (PR) and 10 iCCA recurrent (REC) tumors using data from dataset GSE107102. Pearson correlation coefficients and p-values of correlated genes was reported. In bold genes significantly positively correlated with SERPINB3.

	Gene	PR-Tumors		REC-Tumors	
		Pearson Coefficient	*p value*	Pearson Coefficient	*p value*
**Stem-related Genes**	BMP4	**0,843**	***0,002***	0,345	*0,328*
	BMP7	0,121	*0,740*	0,154	*0,672*
	CD44	**0,655**	***0,040***	**0,736**	***0,015***
	EPCAM	0,287	*0,421*	-0,663	*0,037*
	HNF4A	0,370	*0,293*	-0,547	*0,102*
	HNF4G	0,195	*0,589*	-0,656	*0,039*
	KITLG	**0,659**	***0,038***	0,119	*0,744*
	KLF4	0,327	*0,356*	0,526	*0,118*
	LATS1	0,130	*0,721*	0,586	*0,075*
	LIN28A	0,474	*0,167*	0,544	*0,104*
	MAML1	0,529	*0,116*	-0,297	*0,404*
	MYC	0,502	*0,140*	-0,435	*0,209*
	NOTCH1	-0,545	*0,103*	0,340	*0,336*
	PROM1	0,436	*0,208*	-0,470	*0,170*
	SOX2	0,109	*0,766*	-0,179	*0,621*
	STAT3	-0,075	*0,838*	0,383	*0,275*
	THY1	-0,122	*0,736*	0,114	*0,754*
	YAP1	0,499	*0,142*	-0,182	*0,615*
**EMT and ECM Remodeling-related Genes**	ADAM10	0,126	*0,728*	0,478	*0,162*
	ADAM17	0,469	*0,171*	0,565	*0,089*
	ADAM9	0,187	*0,605*	0,266	*0,457*
	CTNNB1	0,420	*0,227*	0,254	*0,479*
	GPNMB	-0,234	*0,515*	**0,685**	***0,029***
	IL13	-0,499	*0,142*	0,247	*0,492*
	IL13RA1	0,280	*0,434*	-0,040	*0,913*
	IL13RA2	-0,163	*0,652*	**0,865**	***0,001***
	ITGA5	-0,359	*0,308*	0,119	*0,744*
	ITGB1	**0,750**	***0,012***	-0,554	*0,096*
	ITGB3	0,131	*0,718*	0,382	*0,276*
	MMP1	0,477	*0,164*	0,084	*0,817*
	MMP13	0,081	*0,824*	-0,411	*0,238*
	MMP3	0,271	*0,448*	0,006	*0,987*
	MMP7	0,175	*0,629*	-0,610	*0,061*
	SDC1	0,106	*0,771*	-0,672	*0,033*
	SNAI1	-0,199	*0,581*	0,582	*0,078*
	SNAI2	-0,340	*0,337*	0,042	*0,907*
	VIM	-0,100	*0,783*	0,600	*0,067*
	ZEB2	-0,167	*0,644*	**0,711**	***0,021***
