# Supplementary material for: The protease‐inhibitor SerpinB3 as a critical modulator of the stem‐like subset in human cholangiocarcinoma
Source: Liver Int. 2021 Sep 16;42(1):233–48. doi: 10.1111/liv.15049 (PMC9290104; doi:10.1111/liv.15049)
Supplement: Supplementary file 3 — Supporting Information [file LIV-42-233-s002.docx]

LIVint-20-02488 - S

**THE PROTEASE-INHIBITOR SERPINB3 AS A CRITICAL MODULATOR OF THE STEM-LIKE SUBSET IN HUMAN CHOLANGIOCARCINOMA**

**Margherita Correnti^1,2^, Andrea Cappon^3^, Mirella Pastore^4^, Benedetta Piombanti^4^, Giulia Lori^4^, Douglas VPN Oliveira^5^, Patricia Munoz-Garrido^5^, Monika Lewinska^5^, Jesper B Andersen^5^, Cédric Coulouarn^6^, Laurent Sulpice^7^, Caterina Peraldo Neia^8^, Giuliana Cavalloni^9^, Santina Quarta^10^, Alessandra Biasiolo^10^, Matteo Fassan^10^, Matteo Ramazzotti^11^, Matteo Parri^11^, Stefania Recalcati^2^, Luca di Tommaso^12,13^, Claudia Campani^4^, Pietro Invernizzi^14,15^, Guido Torzilli^16^, Fabio Marra^4^, Patrizia Pontisso^10*^, Chiara Raggi^4*#^**

**Table of Contents**

| 1. Supplementary Material and Methods | Page S2 |
| --- | --- |
| 1. Supplementary Figure Legends | Page S9 |
| 1. Supplementary References | Page S12 |
|  |  |
|  |  |
|  |  |
|  |  |
|  |  |
|  |  |

**Supplementary Material and Methods**

**Cell Cultures and Reagents**

HUCCT1, CCLP1 and SG231 cells from intrahepatic bile duct cancer tissues were a kind gift from Dr. A.J. Demetris (University of Pittsburgh, Pittsburgh, PA, US) and were cultured as described previously ^44-46^. The human MTCHC01 cells were cultured as described previously ^47^.

**Cell Transfection**

HUCCT1 and SG231 were stably transfected with a plasmid vector carrying the wild-type SerpinB3 human gene or with the plasmid vector alone (pcDNA3.1D/V5-His-TOPO, Invitrogen Life Technologies), as control. The transfection was carried out using Lipofectamine 3000 reagent (Invitrogen) according to the manufacturer’s instructions. In order to select cells that have acquired the transfected DNA, the complete culture medium was supplemented with 300μg/ml of G418 (Sigma-Aldrich), as a selective marker. ~~HUCCT1~~ Transfected clones were analyzed for SB3 production by real time PCR. Plasmid constructs were obtained as previously described ^18^.

**Sphere Assay**

The cells were grown for 10 days in anchoring-independent conditions with selective serum-free Dulbecco’s modified Eagle’s medium (DMEM)/F12 medium supplemented with 1X B27 supplement minus vitamin A (Life Technologies), human recombinant epidermal growth factor (hrEGF) (R&D System) (20 ng/ml), and bFGF (R&D System) (20 ng/ml). After 7 days, pictures were taken to measure the number and size of CCA-SPH using a Leica DMi1microscope (Leica). Average number of formed spheres microscopic fields (20x) over five fields. CCA-SPH volume was calculated after measuring length 1 (L1) and the length 2 (L2) using the following formula: V = (L1*L2*L2)/2. Each experiment was performed in triplicate. Relative sphere number was expressed as average over the average of MON not pretreated or MON^CTR^ cells ^10^.

**Cell Invasion Assay**

Normal or transfected cells (9 x 10^4^/well) were placed into the upper chamber of a 24-well BioCoatTM Matrigel® Invasion Chamber with a membrane with a 0.4 μm pore size (BD Biosciences) in serum-free RPMI 1640. The cells were allowed to invade toward the lower compartment of the system, which contained either serum-free RPMI 1640 alone or in presence of human recombinant SB3 (rhSB3). After 22 h of incubation at 37°C, the cells that had not penetrated the filter were removed with cotton swabs, and cells that had migrated to the lower surface of the filter were fixed with methanol, stained with Diff Quick solutions, and photographed with an Olympus BX51 microscope with a 20x objective. The values for invasion were expressed as the average number of invaded cells per microscopic field (20x) over five fields. Each experiment was performed in triplicate ^10^.

**Quantitative real-time polymerase chain reaction (RT-PCR)**

Total RNA (500 ng) was transcribed with a High-Capacity cDNA Reverse Transcription Kit (Applied Biosystems). PCR was performed on the cDNA with gene-specific primer pairs (see Table below). Changes in the mRNA expression level of target genes were detected using FAST SYBR-Green PCR Master Mix and the 7900HT Fast Real Time PCR System (Applied Biosystems). The cycling conditions consisted of 20 s at 95°C, 40 cycles of 1 s at 95°C followed by 20 s at 60°C, and a final infinite 4°C hold. The mRNA levels of glyceraldehyde 3-phosphate dehydrogenase (GAPDH) were used for normalization. The differences (2-ΔCt) were calculated using the GAPDH as internal controls. All reactions were performed in triplicate.

| **Gene** | **Primer Forward** | **Primer Reverse** |
| --- | --- | --- |
| **SB3** | **AACTCCTGGGTGGAAAGTCAA** | **ACCAATGTGGTATTGCTGCCAA** |
| **CD13** | **CAGTGACACGACGATTCTCC** | **CCTGTTTCCTCGTTGTCCTT** |
| **CD24** | **TAGGTACCACTATGGGCAGAGCAATGG** | **CCGGAATTCCGTTAAGAGTAGAGATGC** |
| **CD44** | **GTGATGGCACCCGCTATG** | **ACTGTCTTCGTCTGGGATGG** |
| **CD90** | **AGAGACTTGGATGAGGAG** | **CTGAGAATGCTGGAGATG** |
| **CD133** | **GCTTCAGGAGTTTCATGTTGG** | **GGGGAATGCCTACATCTGG** |
| **c-MYC** | **CGGAACTCTTGTGCGTAAGG** | **ACTCAGCCAAGGTTGTGAGG** |
| **NANOG** | **GTCTCGTATTTGCTGCATCG** | **GAAACACTCGGTGAAATCAGG** |
| **NFκB1** | **CTCCGAGACTTTCGAGGAAATAC** | **GCCATTGAAGTTGGTAGCCTTCA** |
| **NOTCH1** | **GCAGTTGTGCTCCTGAAGAA** | **CGGGCGGCCAGAAAC** |
| **STAT3** | **GGCATTCGGGAAGTATTGTCG** | **GGTAGGCGCCTCAGTCGTATC** |
| **YAP** | **ACCCTCGTTTTGCCATGAAC** | **TTGTTTCAACCGCAGTCTCTC** |
| **BMI1** | **TTGCTTTGGTCGAACTTGG** | **GTGCTTCTTTTGCAGACTGG** |
| **BMP4** | **AGCGTAGCCCTAAGCATCAC** | **AGTCATTCCAGCCCACATCG** |
| **KLF4** | **AGACAGTCTGTTATGCACTGTGG** | **TGTTCTGCTTAAGGCATACTTGG** |
| **OCT4** | **TTGTGCCAGGGTTTTTGG** | **ACTTCACCTTCCCTCCAACC** |
| **SOX-2** | **ATGGGTTCGGTGGTCAAGT** | **GGAGGAAGAGGTAACCACAGG** |
| **BCL2L1** | **GACTGAATCGGAGATGGAGACC** | **GCAGTTCAAACTCGTCGCCT** |
| **MMP1** | **CTGGCCACAACTGCCAAATG** | **CTGTCCCTGAACAGCCCAGTACTTA** |
| **MMP2** | **TTGACGGTAAGGACGGACTC** | **ACTTGCAGTACTCCCCATCG** |
| **MMP7** | **GGGATTAACTTCCTGTATGC** | **GATCTCCATTTCCATAGGTTG** |
| **MMP9** | **AAGGATGGGAAGTACTGG** | **GCCCAGAGAAGAAGAAAAG** |
| **MMP13** | **TCCCAGGAATTGGTGATAAAGTAGA** | **CTGGCATGACGCGAACAATA** |
| **ADAM10** | **CTGGCCAACCTATTTGTGGAA** | **GACCTTGACTTGGACTGCACTG** |
| **ADAM12L** | **CACCATTGAAAAACTAAGGTGTGTG** | **GAGCCTGACAGGGTTGGAAG** |
| **ADAM17** | **GAAGGCCAGGAGGCGATTA** | **CGGGCACTCACTGCTATTACC** |
| **ADAM9** | **ACTGTGAAAATGGCTGGGCT** | **TGTATGTAGGTCCACTGTCCACAC** |
| **ITGB3** | **CTCCGGCCAGATGATTC** | **TCCTTCATGGAGTAAGACAG** |
| **SDC1** | **TACTAATTTGCCCCCTGAAG** | **GATATCTTGCAAAGCACCTG** |
| **SDC4** | **CAGGGTCTGGGAGCCAAGT** | **GCACAGTGCTGGACATTGACA** |
| **β-catenin** | **GCTGGGACCTTGCATAACCTT** | **ATTTTCACCAGGGCAGGAATG** |
| **VIM** | **ACACCCTGCAATCTTTCAGACA** | **GATTCCACTTTGCGTTCAAGGT** |
| **SLUG** | **TGCGATGCCCAGTCTAGAAA** | **TTCTCCCCCGTGTGAGTTC** |
| **SNAIL** | **GCTGCAGGACTCTAATCCAGA** | **ATCTCCGGAGGTGGGATG** |
| **GAPDH** | **GATCATCAGCAATGCCTCCT** | **TGTGGTCATGAGTCCTCCCA** |

**RT-PCR array**

The total RNA was extracted with the RNeasy kit (Qiagen) according to the manufacturer’s instructions. The RNA concentration and quality were measured using an optical Nanodrop ND1000 spectrophotometer (Thermo Fisher Scientific).

Total RNA (500 ng) from SPH and MON MTCHC01 cells and in vivo mouse tumors was reverse transcribed into cDNA using an RT² First Strand Kit (SabBioscience, Qiagen) according to the manufacturer’s instructions. RT2 Profiler™ human cancer stem cell (PAHS-1776Z), 384-well PCR arrays (SabBioscience, Qiagen) for MTCHC01 cells and QuantiNova LNA PCR Focus Panels 384-well plates (#SBHS-133ZE, Qiagen) mouse tumors. In each 384-well plate, a number of 3 different tumor samples for each condition were tested for 84 genes specifically associated with liver cancer pathways.

Quantitative PCR were performed by using the ABI ViiA™ 7 System (Applied Biosystems) with the following cycling conditions: 10 min at 95°C, 40 cycles of 15 s at 95°C followed by 1 min at 60°C and a final infinite 4°C hold.

The expression values were calculated with the ΔCt method, using ACTB, GAPDH, RPLP0 and HPRT1 as housekeeping genes as reference. A cutoff of at least 1.5-fold increases and 0.5-fold decreases were considered significant.

Data were centered and normalized, and hierarchical clustering of genes/samples using centered correlation metrics with complete linkage was performed. The grouping of the genes from top to bottom is given for each heatmap**.**

**Western Blot Analysis**

Cells were lysed at 4 °C with lysis buffer (1% Triton X-100, 50 mmol/L Tris–HCl, pH 7.4, 150 mmol/L NaCl, 1 mmol/L EDTA, 1 mmol/L sodium orthovanadate, 2 mmol/L PMSF, and 1 mmol/L each of leupeptin and pepstatin). After 30 min of lysis, cellular extracts were centrifuged for 10 min at 12,000 g, and the supernatant was used for Western blot experiments as detailed previously 25. Antibodies were used for Western blot analysis according to the manufacturer’s recommendations. Immunoblots were incubated overnight at 4 °C with primary antibody in 1% BSA in 1x DPBS. Rabbit polyclonal anti-SerpinB3 (Hepa-Ab, Xeptagen), rabbit polyclonal Anti-Phospho-ERK1/2 (Thr202/Tyr204) (#9101 Cell Signaling Technology), rabbit polyclonal Anti-ERK 1 (K-23) (sc-94 Santa Cruz Biotechnology), rabbit polyclonal Anti-Phospho-p38 (Thr 180/Tyr 182)-R (sc-17852-R Santa Cruz Biotechnology), rabbit polyclonal Anti-p38α (sc-535 Santa Cruz Biotechnology), rabbit polyclonal Phospho-SAPK/JNK (Thr 183/Tyr 185) (#9251 Cell Signaling Technology, Danvers), rabbit polyclonal JNK1 (FL) (sc-571 Santa Cruz Biotechnology), rabbit polyclonal Anti-Phospho- NFkB (Ser 536) (#3031 Cell Signaling Technology), rabbit monoclonal NFkB (#8242 Cell Signaling Technology), rabbit monoclonal β-catenin (#9582 Cell Signaling Technology), rabbit polyclonal anti-MMP9 (Abcam) and mouse monoclonal anti-Notch (Abcam). Immunoblots were then incubated with secondary antibody α-rabbit/mouse (1:4000) in 1% BSA in 1x DPBS for 1 h. Monoclonal Anti-Vinculin antibody produced in mouse (V9131, Sigma) was used as internal control (1:1000) in 1% BSA in 1x DPBS. Quantification of the signal was obtained by chemiluminescence detection on an Image Quant Las4000 (GE Healthcare Life Sciences) and subsequent analysis performed with ImageJ software.

**Gene Silencing**

Control siRNA and SerpinB3 SiRNA (sc-6317) were from Dharmacon. Cells were transfected as previously described using the Amaxa nucleofection technology (Amaxa) according to manufacturer’s instructions.

**Crystal Violet Staining**

CCA cells were plated in 6 well dishes and allowed to adhere overnight. Cells were wash twice with ice cold PBS and then fixed for 10 min in ice cold, 100% methanol. Crystal violet (0.5% in 25% methanol/75% water) was then added for 10 min followed by rinsing the cells in deionized water. Coverslips were dried at room temperature, imaged using an Olympus BX51 microscope with a 20x objective.

**Cell proliferation assay**

Proliferation was evaluated by BrdU incorporation using a colorimetric immunoassay. A total of 7,5 x103 cells were seeded in a 96-well plate and allowed to grow overnight in SPH medium. The Cell Proliferation ELISA-BrdU (colorimetric) Kit (Roche) was used to detect the cell proliferation according to the manufacturer’s protocols.

**Phalloidin staining and Immunocytochemistry**

To investigate the cell morphology, after washing with PBS, the cells were fixed with 3.7% paraformaldehyde solution in PBS for 20 min at 4 °C. Then, after extensive washing in PBS, the cells were permeabilized with 0.1% Triton X-100 in PBS and stained with a 50 μg/ml Phalloidin–Tetramethylrhodamine B isothiocyanate (P1951 Sigma-Aldrich) for 1 h at room temperature followed by several washes with PBS. DAPI (D1306 Thermo Fisher) 300 nM was used to visualize nuclei. The cells were mounted with ProLong™ Gold (Thermo Fisher P36934). All fluorescence samples were examined at room temperature using a microscope (TCS SP8; Leica) with lasers exciting at 405 and 543 nm (Leica) with a 40x objective. PMT levels were set using control samples. Multicolor images were collected simultaneously in two channels. Images were taken using a 63x, 1.4–0.6 NA, oil, HCX Plan APO lens. Images were captured using the Leica LASX image acquisition software. Overlays were generated using LASX software. Photo montages were generated using LASX software.

**Immunofluorescence**

Expression of SB3, MMP9, CD44 and NOTCH immunofluorescence was performed on O.C.T. embedded tumors obtained from NSG mice after injection of 1,000 sphere- or monolayer-derived SerpinB3 transfected and control HUCCT1 cells. The cryostat sections on slide were fixed with Merckofix spray (Merck Millipore), permeabilized with 0.2% Triton X 100 and blocked with 5% Goat serum in 1% BSA in PBS. Slides were then incubated with the primary antibodies anti-SB3 (polyclonal; rabbit; Hepa-Ab, Xeptagen), anti-MMP9 (polyclonal; rabbit; Abcam) and anti-Notch (monoclonal; mouse; Abcam) and then with 546 Alexa Fluor anti-rabbit antibody and 488 Alexa Fluor anti-rabbit secondary antibody, respectively (Invitrogen Life Technologies). Anti-CD44 (monoclonal; rat; APC-eFluor 780; Invitrogen Life Technologies).

Cell nuclei were counterstained with DAPI (Sigma-Aldrich). Slides were mounted with ELVANOL (Sigma-Aldrich) and observed under fluorescence microscope using the optical sectioning of Apotome.2 (Axiovert 200M, Carl Zeiss MicroImaging GmbH). Fluorescence quantification analysis was performed using the Axiovision software and the fluorescence extent of each molecule was normalized by nuclei content, represented by DAPI staining.

**Supplementary Figure Legends**

**Supplementary Figure 1.** qRT-PCR arrays focused on cancer stem cells pathways. Heatmap of MTCHC01 monolayer (MON) and spheres (SPH) samples based on expression of 84 genes. Gene expression levels are expressed in color code from green (low) to red (high) according to the color key scale bar. Hierarchical clustering was based on complete linkage on euclidean distances between genes (rows) or samples (columns).

**Supplementary Figure 2.** Experimental design of paracrine and endogenous SB3 effect on CCA cells.

**Supplementary Figure 3.** Global characterization of paracrine SB3 effect (+rhSB3) in MTCHC01 cells. A) Expression of SerpinB3 (SB3) mRNA in MTCHC01 cells after exogenous addition of rhSB3. GAPDH was used as the internal control. Not treated monolayer (MON) as control. All mRNA levels are presented as fold changes normalized to 1 (mean expression of MON).  Data are expressed as mean ± SEM (p value vs. MON by Student t test, *p ≤0.05, **p ≤0.01, *** p≤0.001). B) Spheres generated after 6-days-treatment with SB3 (+rhSB3) were analyzed using low adherent 96-wells. Spheres numbers/field were counted and normalized to sphere numbers generated by MON condition (n = 3). Mean ± SEM (p value vs. MON by Student’s t test, *p ≤0.05, **p ≤0.01, *** p≤0.001). C) Left: SB3 paracrine effect on MTCHC01-SPH volume. Results normalized to SPH volume related to MON condition (n = 3). Mean ± SEM (p value vs. MON by Student’s t test, *p ≤0.05, **p ≤0.01, *** p≤0.001). Right: Representative images of MTCHC01 SPH are shown (original magnification 40X, scale bar 50μM). D) Invasion assay using Matrigel-coated transwells. MTCHC01 cells counted and normalized to migrated not pretreated monolayer cells (MON), as control (n = 3). Mean ± SEM (p value vs. MON by Student’s t test, *p ≤0.05, **p ≤0.01, *** p≤0.001). E) Relative expression of transcript-encoding liver CSC-like, Self-Renewal, Pluripotency, Epithelial Mesenchymal Transition (EMT) as well as Extracellular Matrix (ECM) remodeling genes in SB3-treated (+rhSB3) MTCHC01 cells. GAPDH was used as an internal control. Not treated monolayer cells (MON) as control. All mRNA levels are presented as fold changes normalized to 1 (mean expression of MON). Data are expressed as Mean ± SEM (p value vs. MON by Student t test, * p≤0.05, **p ≤0.01, *** p≤0.001). F) SB3 paracrine effect on morphology of MTCHC01 cells highlighted by Crystal Violet (left) and Phalloidin (right) staining. Representative images are shown (original magnification 20X for Crystal Violet and 40x for Phalloidin).

**Supplementary Figure 4.** A) HUCCT1 and SG231 spheres generated after 6-days-treatment (+rhSB3) or transfection (MON^SB3+^) with SB3 were analyzed using low adherent 96-wells. Spheres numbers/field were counted and normalized to sphere numbers generated by MON or MON^CTR^ conditions (n = 3). Mean ± SEM (p value vs. MON or MON^CTR^, respectively, by Student’s t test, *p ≤0.05, **p ≤0.01, *** p≤0.001). B) Representative images of HUCCT1 and SG231 invaded cells are shown (original magnification 20X, scale bar 100μM). C and D) SB3 paracrine and endogenous effect on morphology of HUCCT1 and SG231 cells highlighted by Crystal Violet (C) and Phalloidin (D) staining. Representative images are shown (original magnification 20X for Crystal Violet and 40x for Phalloidin).

**Supplementary Figure 5.** Tumor volume growth curves of the mice after injection of 1000 HUCCT1 SPH, MON^SB3+^ and MON^CTR^ isolated cells. The difference in tumor volume growth among the MON^SB3+^ and MON^CTR^ groups was statistically significant. Mean ± SEM (n=6; p value vs. MON^CTR^ by Student’s t test, * p≤0.05, **p ≤0.01, *** p≤0.001).

**Supplementary Figure 6.** A) Effects of SerpinB3 silencing in CCA SPH cells (SPH^SB3-^) on sphere forming ability. Spheres generated were analyzed using low adherent 96-wells. Spheres numbers/field were counted and normalized to sphere numbers generated by SPH^CTR^ condition (n = 3). Mean ± SEM (p value vs. SPH^CTR^ by Student’s t test, *p ≤0.05, **p ≤0.01, *** p≤0.001). B) Representative images of SPH^CTR^ and SPH^SB3-^ invaded cells are shown (original magnification 20X, scale bar 100μM).

**Supplementary Figure 7.** Overall survival (OS, 5 years) for 110 CCA patients from EGA00001000950 database sub-grouped based on SB3 expression. Patients were stratified with an average signal of 0.1 RPKM as the cut off value. Samples <0.1 RPKM would be “no/low expression” and samples >0.1 RPKM would be “high”. Overall survival (OS) was demonstrated by Kaplan-Meier and Log-rank statistics.

**Supplementary References**

44. Miyagiwa M, Ichida T, Tokiwa T, Sato J, Sasaki H. A new human cholangiocellular carcinoma cell line (HuCC-T1) producing carbohydrate antigen 19/9 in serum-free medium. In Vitro Cell Dev Biol 1989;25(6):503-10.

45. Han C, Wu T. Cyclooxygenase-2-derived prostaglandin E2 promotes human cholangiocarcinoma cell growth and invasion through EP1 receptor-mediated activation of the epidermal growth factor receptor and Akt. J Biol Chem 2005;280(25):24053-63.

46. Shimizu Y, Demetris AJ, Gollin SM, Storto PD, Bedford HM, Altarac S, Iwatsuki S, Herberman RB, Whiteside TL. Two new human cholangiocarcinoma cell lines and their cytogenetics and responses to growth factors, hormones, cytokines or immunologic effector cells. Int J Cancer 1992;52(2):252-60.

47. Cavalloni G, Peraldo-Neia C, Varamo C, Casorzo L, Dell'Aglio C, Bernabei P, Chiorino G, Aglietta M, Leone F. Establishment and characterization of a human intrahepatic cholangiocarcinoma cell line derived from an Italian patient. Tumour Biol 2016;37(3):4041-52.
